# Efficacy and safety of herbal formulas with the function of gut microbiota regulation for gastric and colorectal cancer: A systematic review and meta-analysis

**DOI:** 10.3389/fcimb.2022.875225

**Published:** 2022-08-04

**Authors:** Bowen Xu, Xinmiao Wang, Heping Wang, Luchang Cao, Yuansha Ge, Bo Yuan, Ruike Gao, Jie Li

**Affiliations:** ^1^ Department of Oncology, Guang’anmen Hospital, China Academy of Chinese Medical Sciences, Beijing, China; ^2^ Graduate School, Beijing University of Chinese Medicine, Beijing, China; ^3^ Department of Rheumatology, Guang’anmen Hospital, China Academy of Chinese Medical Sciences, Beijing, China

**Keywords:** herbal formulas, gut microbiota, gastric cancer, colorectal cancer, meta-analysis, efficacy, safety

## Abstract

**Background:**

Currently, gastric cancer (GC) and colorectal cancer (CRC) are the most common causes of cancer-related mortality worldwide. Gut microbiota is closely related to the occurrence of GC and CRC and the efficacy of chemotherapy. This study is aimed at evaluating the efficacy and safety of herbal formulas with the function of gut microbiota regulation (HFGMR) in the treatment of GC and CRC and to assess the quality of the synthesized evidence.

**Methods:**

A comprehensive search was performed on eight electronic databases, PubMed, EMBASE, CENTRAL, Web of Science, Chinese Biomedical Literature Database, China National Knowledge Infrastructure, Wanfang database, Chinese Scientific Journals Database, and two registries, Chinese Clinical Trial Registry and ClinicalTrials.gov, from their initiation to January 2022. Randomized controlled trials (RCTs) studying the therapeutic effects of HFGMR were included. We used Stata 16 for data synthesis and Risk of Bias 2 (RoB 2) for methodological quality evaluation and assessed the quality of the synthesized evidence in the Grading of Recommendations, Assessment, Development and Evaluations (GRADE) approach.

**Results:**

Fifty-three RCTs involving 4,478 patients were included. These trials involve seven herbal formulas that could regulate the gut microbiota of *Bifidobacterium*, *Lactobacillus*, *Escherichia coli*, *Bacteroides*, and *Enterococcus faecalis*. The meta-analysis results were subgrouped to three different stages in GC and CRC. 1) For the perioperative stage, HFGMR combined with conventional therapy could shorten the time to bowel sound recovery by 1.63 h [mean difference (MD) = −1.63, 95% confidence interval (CI) (−2.62, −0.65)], the time to first flatus by 9.69 h [MD = −9.69, 95% CI (−10.89, −8.48)], and the duration of hospitalization by 2.91 days [MD = −2.91, 95% CI (−4.01, −1.80)] in GC. There were no significant differences in outcomes of gastrointestinal function recovery and adverse events in CRC. 2) For postoperative patients, combined with adjuvant chemotherapy, HFGMR could decrease the incidence of diarrhea, nausea and vomiting, anorexia, and peripheral neurotoxicity in GC; boost Karnofsky performance status (KPS) improvement rate [risk ratio (RR) = 1.96, 95% CI (1.38, 2.79)]; and decrease the incidence of leucopenia and nausea and vomiting in CRC. 3) For advanced stage, HFGMR can significantly improve the objective response rate (ORR) [RR = 1.35, 95% CI (1.19~1.53)], disease control rate (DCR) [RR = 1.14, 95% CI (1.05~1.23)], and KPS improvement rate [RR = 1.56, 95% CI (1.17, 2.09)] and decrease the incidence of leucopenia, neutropenia, anemia, nausea and vomiting, diarrhea, and fatigue in GC. There were no significant differences in ORR [RR = 1.32, 95% CI (0.94~1.86)] and DCR [RR = 1.22, 95% CI (0.99~1.50)], but they can improve the KPS response rate [RR = 1.62, 95% CI (1.13, 2.32)] and decrease the incidence of myelosuppression, nausea and vomiting, diarrhea, and hepatic and renal dysfunction in CRC.

**Conclusion:**

This study indicates that herbal formulas that could regulate the composition and proportion of gut microbiota have a positive effect in three stages (perioperative, postoperative, and advanced) of GC and CRC. They could promote the recovery of postoperative gastrointestinal function, increase tumor response, improve performance status, and reduce the incidence of adverse events. Herbal formulas exerted anti-cancer efficacy through multiple mechanisms and pathways; among them, the regulation of gut microbiota has not been paid enough attention. To further support the conclusion and better understand the role of gut microbiota in the treatment of GC and CRC, more rigorously designed, large-scale, and multicenter RCTs that focus on herbal formulas and gut microbiota are needed in the future.

## 1 Introduction

Gastric cancer (GC) and colorectal cancer (CRC), as the main gastrointestinal cancers, are common malignant cancers with high morbidity and mortality. According to the latest global cancer data published by *JAMA Oncology* (Kocarnik et al., 2021), the number of newly diagnosed CRC and GC patients in 2019 was 2,170,000 and 1,270,000, respectively, which leaves an unsolved health problem that affects people all over the world. Notably, studies have reported that gastrointestinal cancers are closely related to gut microbiota (Weng et al., 2019; Zhou et al., 2021), a large microbial community known as the ‘second gene’ existing in the intestinal tract that has a significant effect. Dysbiosis of gut microbiota can result in the production of carcinogenic bacteria (Fan et al., 2021), such as *Bacteroides fragilis*, *Enterococcus faecalis*, and *Helicobacter hepaticus* (Meng et al., 2018). These carcinogenic bacteria can secrete toxins, cause intestinal cell damage, and induce gastrointestinal cancers (Mármol et al., 2017; Cheng et al., 2020; Overacre-Delgoffe et al., 2021). On the contrary, the healthy gut microbiota can inhibit the growth of conditioned pathogenic bacteria and form a protective barrier to regulate gastrointestinal inflammation and immunity (Amoroso et al., 2020). Interestingly, it was reported that the transplantation of healthy fecal microbiota can prevent intestinal injury in CRC (Chang et al., 2020). Therefore, it is important to make a special effort to keep a healthy state of gut microbiota in the treatment of gastrointestinal cancers.

Traditional Chinese herbal medicine has a long history of treating diseases by regulating gut microbiota. As early as 1,400 years ago, ‘purified feces’ was mentioned in the book, ‘Lei’s Treatise on Preparing Drugs’. The ‘purified feces’ was produced by repeatedly washing the feces of healthy people with well or underground spring water and then filtering and burying them underground for at least 1 year. It is the earliest record of traditional Chinese herbal treatment for fecal microbiota transplantation. With the development of modern technology, studies have verified that herbal formulas have a good effect on the regulation of gut microbiota. For example, Gegenqinlian decoction can regulate intestinal mucosal immunity and glucolipid metabolism by enriching butyric-producing bacteria, thus reducing systemic and local pancreatic inflammation and improving insulin resistance (Xu et al., 2020). Ginseng extract can enrich *E. faecalis* and promote the production of unsaturated long-chain fatty acid-nutmeg oleic acid, which can stimulate the thermogenic activity of brown fat, induce the formation of beige fat, reduce fat accumulation, and improve obesity (Quan et al., 2020). Apart from metabolic diseases, Gegenqinlian decoction can enhance the effect of PD-1 blockade in CRC with microsatellite stability by remodeling the gut microbiota and the tumor microenvironment (Lv et al., 2019), indicating that herbal formulas can also treat gastrointestinal cancers by regulating the gut microbiota. With the growing number of studies on the value of herbal formulas in gastrointestinal cancer treatment, more randomized controlled trials (RCTs) have been published in recent years. This study is aimed to seek effective strategies for the treatment of gastrointestinal cancers through a systematic review and meta-analysis of herbal formulas with the function of gut microbiota regulation (HFGMR).

## 2 Methods

This study was performed under the guidance of the Preferred Reporting Items for Systematic Reviews and Meta-Analyses (PRISMA) statement and checklist (Moher et al., 2009); see PRISMA checklist in **Supplementary Material 1**. This study was registered on PROSPERO (No. CRD42021292096).

### 2.1 Eligibility criteria

#### 2.1.1 Type of studies

This study included RCTs and observational studies, and quasi-RCTs were excluded. Trials that did not describe the randomization method in detail were considered non-randomized studies of interventions and were excluded. Animal studies were also excluded.

#### 2.1.2 Types of participants

RCTs in which participants were diagnosed with GC or CRC through cytological or pathological tests were included.

#### 2.1.3 Types of intervention and control

Randomized studies of herbal formulas with the function of gut microbiota regulation as the sole treatment or combined with other treatments were included in this study.

#### 2.1.4 Types of outcomes

RCTs reporting outcomes related to clinical efficacy and safety of herbal formulas in gastrointestinal cancer treatment were included in this study. Trials that only reported outcomes of laboratory test results were excluded.

For perioperative patients, the outcomes of gastrointestinal function recovery, duration of hospitalization, time to first oral feeding, and time to out-of-bed activity were included; for postoperative or advanced patients, the outcomes of long-term survival and tumor response rate (TRR) of anti-cancer treatment were included; quality of life (QoL), performance status (PS), and incidence of adverse events (AEs) as safety outcomes were included for patients of all stages.

### 2.2 Selection of herbal formulas with the function of gut microbiota regulation

We performed a preliminary search to select the herbal formulas with the function of gut microbiota regulation. In order to locate appropriate herbal formulas that could regulate gut microbiota in patients with GC or CRC, we searched eight electronic databases with a search strategy based on the keywords ‘herbal medicine’, ‘Chinese medicine’, ‘Kampo medicine’, ‘gut microbiota’, ‘gastric cancer’, and ‘colorectal cancer’. We obtained 1,431 records from database searches, and nine formulas were located after our rigorous selection. These nine formulas were included as search terms in the further search for RCTs.

### 2.3 Search strategy

We searched PubMed, EMBASE, CENTRAL, Web of Science, the Chinese Biomedical Literature Database (CBM), the China National Knowledge Infrastructure (CNKI), the Wanfang database, and the Chinese Scientific Journals Database (VIP database). Searches were performed from the database initiation to January 2022. The language restriction was English and Chinese. We also searched the Chinese Clinical Trial Registry (ChiCTR) and ClinicalTrials.gov to identify ongoing and completed trials. The search strategy was based on the combination of controlled vocabulary (MeSH terms and Emtree terms) and free-text terms. The terms ‘Stomach Neoplasms’, ‘Colorectal Neoplasms’, ‘Colonic Neoplasms’, ‘Rectal Neoplasms’, ‘si-jun-zi-tang’, ‘gegenqinlian’, ‘liu-jun-zi-tang’, ‘xiaochaihu’, ‘shosaiko-to’, ‘danggui buxue decoction’, ‘shenling baizhu san’, ‘dai-kenchu-to’, ‘jishengwumeiwan’, and ‘quxie capsule’ were used to develop the search strategy for PubMed, which is shown in **Supplementary Material 2**. Modifications to the search strategy were used with other databases.

### 2.4 Screening and selection

Search results were imported to EndNote 20. Two authors (HW and BX) reviewed the titles and abstracts in the database search results after duplicate removal. The full texts of potential articles were then reviewed and assessed for their eligibility. Screening and selection were independently processed in duplicate by the two reviewers (HW and BX). RCTs that met the inclusion criteria were included. The process is summarized using a PRISMA flow diagram.

### 2.5 Data extraction

The following data were extracted from the included studies: 1) identification information (first author and year of publication), 2) general information (study setting, sample size, and duration of follow-up), 3) participants (clinical stage, age, and sex), 4) intervention details (name of herbal formulas, dose, frequency, and duration), 5) comparison details (name, dose, frequency, and duration of treatment), and 6) outcome details. The authors of the trials were contacted for any missing or incomplete data.

### 2.6 Quality assessment

The Risk of Bias 2 (RoB 2) tool was used to assess the methodological quality of included studies (Sterne et al., 2019). We evaluated outcomes of included studies of the risk of bias of the randomization process, deviation from intended intervention, missing outcome data, outcome measurement, and selection of the reported result; the overall quality of RCTs was evaluated as low, with some concerns or high risk of bias.

### 2.7 Evidence synthesis for randomized controlled trials

Stata 16 was used in data synthesis to perform a meta-analysis. The mean differences (MDs) for continuous data and risk ratio (RR) for dichotomous data with 95% confidence intervals (CIs) were evaluated. The random-effects model was used when synthesizing data for the meta-analysis. We quantified inconsistency by applying the I^2^ statistic; a value of I^2^ > 50% was considered substantial heterogeneity, and I^2^ > 75% was considered heterogeneity (Higgins et al., 2019). Subgroup analyses were performed according to the different treatments that patients received in control groups and to explore the source of heterogeneity if substantial heterogeneity existed. A meta-analysis was precluded in some conditions (limited evidence for comparison, existence of considerable heterogeneity, or different effect measures) (Higgins et al., 2019), and descriptive analysis was used in these conditions.

Given the strong correlation between the two anti-tumor treatment response evaluation criteria, WHO criteria, and Response Evaluation Criteria in Solid Tumors (RECIST) criteria, the outcomes reported by these two criteria were considered homogeneous (Aras et al., 2016).

Publication bias of the cumulative evidence among individual studies was evaluated using a graphical method of funnel plot and Egger’s test (Egger et al., 1997) if at least 10 studies were included for the synthesized outcome.

### 2.8 Quality of evidence

The quality of the cumulative evidence was evaluated using the Grading of Recommendations, Assessment, Development, and Evaluations (GRADE) system (Guyatt et al., 2008). The risk of bias, inconsistency, indirectness, imprecision, and publication bias were evaluated. The quality of evidence was classified as high, moderate, low, or very low quality (Guyatt et al., 2008). We presented our findings in a summary of findings (SoF) table.

## 3 Results

**Figure 1:**
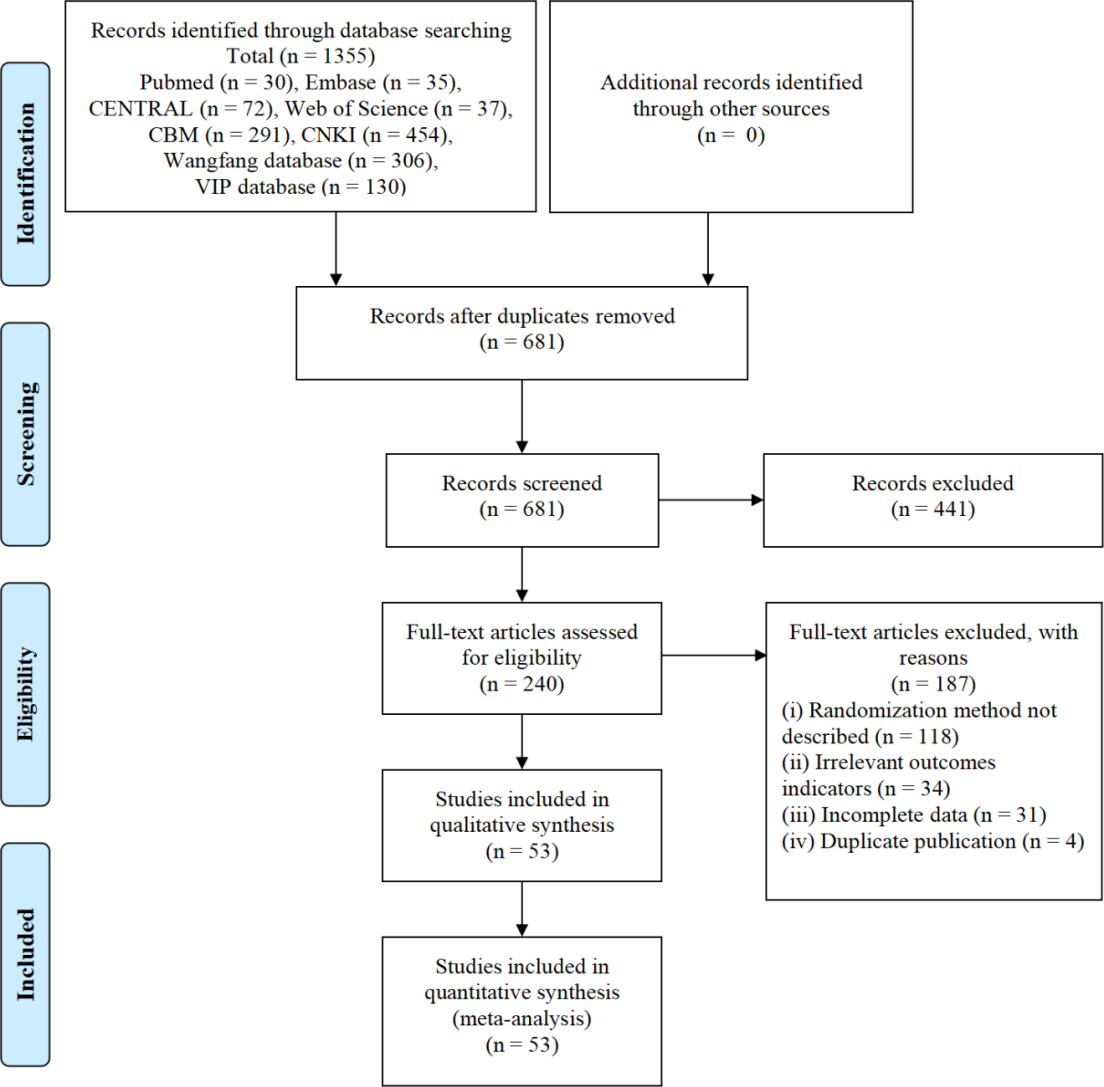
Flowchart of the selection process.

We obtained 1,355 records from database searches, and after the selection process, there were 53 trials involving 4,478 participants included in this SR. The selection process was summarized as a flowchart shown in **Figure 1**.

### 3.1 Details of included trials

Among these 53 trials, there are five double-blinded RCTs, and four of them were conducted in multicenter. The sample size of included trials ranged from 17 to 336. Most of the included trials were RCTs of a small sample size conducted in a single center. The details are shown in **Table 1**.

**Table 1:** Study characteristics of included RCTs.

Study	Study design	Sample size		Age		Sex (M/F)		Stage (Ⅰ/Ⅱ/Ⅲ/Ⅳ/UK)		Intervention		Outcomes
		T	C	T	C	T	C	T	C	T	C	
Perioperative GC												
Yoshikawa et al. (2015)	Multicenter, double-blinded, placebo-controlled	96	99	68 (33–83)	67 (28–84)	73/23	76/23	29/26/31/10	20/31/43/4/1	Daikenchuto + SC	SC	①②⑤⑥
Akamaru et al. (2015)	Multicenter	41	40	63.4 ± 8.9	63.7 ± 9.2	31/10	27/13	26/6/9/0	20/8/12/0	Daikenchuto + SC	SC	①⑤⑥
Chen et al. (2020)	Single-center	40	44	58.9 ± 15.1	59.4 ± 13.9	21/19	24/20	1/17/22/0	2/18/24/0	LJZ + ERAS	ERAS	①②
Fu et al. (2007)	Single-center	40	40	52.8 ± 5.5	53.6 ± 5.2	15/25	14/26	–	–	SJZ + SC	SC	①
Huang et al. (2018)	Single-center	30	30	55.67 ± 8.26	57.3 ± 8.71	18/12	17/13	0/10/17/3	0/8/20/2	LJZ + ERAS	ERAS	①
Yue et al. (2019)	Single-center	40	40	54.98 ± 5.86	55.13 ± 6.04	28/12	26/14	–	–	LJZ + SC	SC	①⑥
Li (2020)	Single-center	55	55	41.27 ± 5.62	42.93 ± 5.48	31/24	29/26	–	–	SJZ + EN	EN	①
Yu and Ren (2019)	Single-center	45	45	51.50 ± 5.74	52.75 ± 5.38	29/16	28/17	9/22/14/0	8/24/13/0	SJZ + EN	EN	①
Cao et al. (2020)	Single-center	30	30	53.79 ± 8.62	53.33 ± 8.28	20/10	21/9	–	–	LJZ + ERAS	ERAS	①②⑤
Postoperative GC												
Li et al. (2020a)	Single-center	56	56	53.04 ± 6.12	53.65 ± 4.98	30/26	34/22	0/0/34/22	0/0/31/25	SLBZ + FOLFOX	FOLFOX adjuvant chemotherapy	⑤⑥
Li (2017)	Single-center	38	38	59.37 ± 3.24	59.56 ± 3.17	16/22	23/15	–	–	SLBZ + FOLFOX	FOLFOX adjuvant chemotherapy	③⑤⑥
Advanced GC												
Lai et al. (2018)	Single-center	30	30	45	44	22/8	24/6	0/0/0/30	0/0/0/30	SLBZ + 5-FU + CF + TAX	5-FU + CF + TAX chemotherapy	⑤⑥
Jia and Zhang (2017)	Single-center	59	59	61.93 ± 5.22	62.27 ± 5.16	29/30	31/28	Advanced	Advanced	SLBZ + 5-FU + DP	5-FU + DP chemotherapy	④⑥
Zhang and Su (2008)	–	36	36	55 (30–65)	54 (26–63)	24/12	23/13	0/0/15/21	0/0/16/20	SLBZ + 5-FU + CF + DDP	5-FU + CF + DDP chemotherapy	⑤⑥
Li et al. (2016)	Single-center	50	50	62.16 ± 1.17	58.68 ± 1.25	29/21	26/24	Advanced	Advanced	SLBZ + TS	TS chemotherapy	④⑤⑥
Wang et al. (2014)	Single-center	45	45	61.5 ± 1.0	62.0 ± 1.0	27/18	26/19	Advanced	Advanced	SLBZ + FOLFOX	FOLFOX chemotherapy	④⑤⑥
Zhong et al. (2019)	Single-center	41	41	54.86 ± 3.77	55.04 ± 3.14	22/19	20/21	Advanced	Advanced	SLBZ + FOLFOX	FOLFOX chemotherapy	④⑤⑥
Shen et al. (2021)	Single-center	34	34	56.46 ± 8.37	56.37 ± 8.53	18/16	19/15	0/0/23/11	0/0/22/12	LJZ + ECF	ECF chemotherapy	③⑤⑥
Wang and Yan (2020)	Single-center	39	39	68.5 ± 8.0	68.7 ± 8.2	20/19	22/17	Advanced	Advanced	LJZ + TXELOX	TXELOX chemotherapy	⑤
Lin et al. (2017)	Single-center	35	34	53 (32–70)	51 (30–68)	20/15	18/16	0/0/13/22	0/0/20/14	LJZ + 5-FU + CF + TAX	5-FU + CF + TAX chemotherapy	④⑤⑥
Li et al. (2010)	Single-center	42	40	58 (31–69)	55 (32–70)	22/20	21/19	Advanced	Advanced	LJZ + 5-FU + CF + TAX	5-FU + CF + TAX chemotherapy	④⑤⑥
Wang et al. (2019)	Single-center	30	30	70.53 ± 3.96	72.17 ± 3.98	16/14	17/13	0/0/0/30	0/0/0/30	SJZ + Apatinib	Apatinib	④⑤⑥
Xie and Chen (2020)	Single-center	56	56	57.26 ± 2.14	57.29 ± 2.11	22/34	23/33	0/8/30/8	0/10/40/6	LJZ + S-1	S-1 chemotherapy	④⑥
Perioperative CRC												
Katsuno et al. (2015)	Multicenter, double-blinded, placebo-controlled	174	162	68 (28–88)	69 (35–91)	98/76	99/63	–	–	Daikenchuto + SC	SC	①
Katsuno et al. (2016)	Multicenter, double-blinded, placebo-controlled	38	33	67.7 (39–88)	68.2 (51–85)	17/21	20/13	2/21/15/0	1/18/14/1	Daikenchuto + SC	SC	①⑥
Yaegashi et al. (2014)	Single-center	26	25	69 (51–83)	68 (43–89)	15/11	10/15	–	–	Daikenchuto + SC	SC	①②
Hanada et al. (2021)	Single-center	8	9	63 (55–73.8)	64 (58–67)	4/4	6/3	6/1/1/0	3/0/2/4	Daikenchuto + SC	SC	①②⑤⑥
Wakasugi et al. (2020)	Single-center	16	16	68 (59–79)	68 (44–78)	10/6	10/6	5/6/5/0	7/3/6/0	Daikenchuto + SC	SC	①⑥
Zhang (2020)	Single-center	34	35	67.53 ± 8.89	65.31 ± 11.02	18/16	15/20	–	–	LJZ + ERAS	ERAS	①②
Yang (2021)	Single-center	55	55	56.60 ± 6.79	55.96 ± 6.36	34/21	33/22	–	–	SJZ + EN	EN	①⑥
Chen (2013)	Multicenter	30	30	59.1 ± 10.11	56.86 ± 8.23	19/11	16/14	–	–	LJZ + EN	EN	①②
Postoperative CRC												
Bai et al. (2017)	Single-center	45	44	62.87 ± 12.99	60.18 ± 12.23	26/19	24/20	Dukes B:19 Dukes C: 26	Dukes B: 15 Dukes C: 29	SLBZ + FOLFOX4	FOLFOX4 adjuvant chemotherapy	⑤⑥
Wang and Liu (2020)	Single-center	42	42	59.04 ± 11.12	58.73 ± 10.69	24/18	23/19	0/29/13/0	0/28/14/0	SLBZ + FOLFOX4	FOLFOX4 adjuvant chemotherapy	⑤⑥
Wang et al. (2018)	Single-center	32	32	61.3 ± 5.4	60.8 ± 6.1	18/14	17/15	0/4/28/0	0/7/25/0	DGBX + FOLFOX6	FOLFOX6 adjuvant chemotherapy	⑤⑥
Sun (2020)	Single-center	39	38	58.13 ± 7.12	59.89 ± 5.01	20/19	21/17	0/9/19/11	0/8/21/9	GGQL + mFOLFOX6	mFOLFOX6 adjuvant chemotherapy	⑤⑥
Dong (2018)	Single-center	45	45	53.70 ± 4.16	53.64 ± 4.14	27/18	29/16	Dukes A: 21 Dukes B: 24	Dukes A: 23 Dukes B: 22	SJZ + FOLFOX4	FOLFOX4 adjuvant chemotherapy	⑤⑥
Lim (2014)	Multicenter	30	30	55.40 ± 9.90	49.50 ± 11.29	14/16	19/11	0/5/11/14	0/3/18/9	SJZ + adjuvant chemotherapy	Adjuvant chemotherapy	⑤⑥
Liu et al. (2019)	Single-center	60	60	56.14 ± 5.33	55.68 ± 5.17	32/28	34/26	–	–	SJZ + FOLFOX7	FOLFOX7 adjuvant chemotherapy	⑤
Tong et al. (2017)	Single-center	39	39	64(35-74)	63(49-74)	21/18	23/16	0/11/28/0	0/17/22/0	LJZ + adjuvant chemotherapy	Adjuvant chemotherapy	⑤
Yang (2015)	Single-center	48	48	58.32 ± 11.03	60.53 ± 12.67	–	–	–	–	QXC following adjuvant chemotherapy	Blank control	③
Yang et al. (2007)	Double-center, double-blinded, placebo-controlled	23	21	55.24 ± 29.38	52.4 ± 26.72	14/9	14/7	0/15/8/0	0/13/8/0	QXC following adjuvant chemotherapy	Placebo	③⑤
Yue (2016)	Single-center	65	65	56.42 ± 6.82	55.86 ± 7.33	36/29	38/27	Dukes A: 26 Dukes B: 39	Dukes A: 31 Dukes B: 34	SJZ + FOLFOX7	FOLFOX7 adjuvant chemotherapy	⑤
Liu and Xia (2019)	Single-center	45	45	58.23 ± 4.21	57.31 ± 4.21	27/18	25/20	0/22/23/0	0/25/20/0	LJZ + mFOLFOX6	mFOLFOX6 adjuvant chemotherapy	⑤
Advanced CRC												
Liu (2016)	Single-center	30	30	51.83 ± 14.04	51.03 ± 10.81	17/13	22/8	0/0/4/26	0/0/3/27	SLBZ + chemotherapy	Chemotherapy	⑤⑥
Nan and Li (2016)	Single-center	23	25	61.0 ± 1.0	56.0 ± 1.0	15/8	11/14	Advanced	Advanced	SLBZ + CPT-11+RTX	CPT-11+RTX Chemotherapy	④⑤⑥
Yang et al. (2019)	Single-center	21	20	70-80-16, 80-5	70-80-13, 80-7	12/9	10/10	Advanced	Advanced	DGBX + Xeloda	Xeloda	③④⑥
Zhang et al. (2018)	Single-center	31	28	63.19 ± 10.60	61.36 ± 10.58	17/14	19/9	Advanced	Advanced	LJZ + RTX-based chemotherapy	RTX-based chemotherapy	④⑤⑥
Yang et al. (2008)	Single-center	18	19	63.05 ± 11.17	62.35 ± 11.42	10/8	8/11	Advanced	Advanced	QXC+ chemotherapy	Chemotherapy	③⑤
Zhang et al. (2021a)	Single-center, double-blinded, placebo-controlled	30	30	≤65:10 <65:20	≥65:14 <65:16	13/17	24/6	Advanced	Advanced	QXC+ standard treatment	Standard treatment	③
Jia and Dong (2019)	Single-center	52	54	62.54 ± 10.17	64.73 ± 11.25	31/21	35/19	0/0/23/29	0/0/28/26	SJZ + mFOLFOX6	mFOLFOX6 chemotherapy	④⑤⑥
Xia et al. (2021)	Single-center	60	60	53.99 ± 5.33	54.45 ± 5.21	34/26	33/27	0/0/38/22	0/0/37/23	SJZ + FOLFOX6	FOLFOX6 chemotherapy	④⑤
Xue et al. (2021)	Single-center	40	40	50.48 ± 18.42	50.58 ± 18.52	27/13	26/14	0/0/40/0	0/0/40/0	SJZ + FOLFOX6	FOLFOX6 chemotherapy	⑤⑥
Wang and Zhang (2018)	Single-center	40	40	72.14 ± 3.12	71.32 ± 3.58	21/19	18/22	Advanced	Advanced	SLBZ + XELOX	XELOX chemotherapy	④⑤⑥

① Gastrointestinal function recovery outcomes, ② duration of hospitalization, ③ long-term survival outcomes, ④ response evaluation of anti-cancer treatment, ⑤ quality of life and performance status, and ⑥ AE. M, male; F, female; T, treatment; C, control; ERAS, enhanced recovery after surgery; SC, supportive care; EN, enteral nutrition; LJZ, Liujunzi Decoction; SJZ, Sijunzi Decoction; SLBZ, Shenlingbaizhu powder; DGBX, Danggui Buxue Decoction; QXC, Quxie Capsule; GGQL, Gegenqinlian Decoction; AE, adverse event.

#### 3.1.1 Intervention details

The intervention of treatment included seven formulas that had been verified with the function of regulating gut microbiota in clinical studies. These seven herbal formulas could regulate the composition and proportion of gut microbiota. *Bacteroides*, *Bifidobacterium*, *Lactobacillus*, *E. faecalis*, and *Escherichia coli* were the most reported gut microbiota regulated by these herbal formulas. The function of gut microbiota regulation in these seven formulas is shown in **Table 2**. A total of 14 trials evaluated the efficacy and safety of Liujunzi decoction and modified Liujunzi decoction in GC and CRC, 13 trials evaluated the efficacy and safety of Shenlingbaizhu powder and modified formulas of Shenlingbaizhu in GC and CRC, 12 trials evaluated the efficacy and safety of Sijunzi decoction and modified Sijunzi decoction in GC and CRC, 7 trials evaluated the efficacy and safety of Kampo herbal medicine Daikenchuto (known as Dajianzhong decoction in Chinese medicine) in perioperative patients with GC or CRC, 4 trials evaluated the efficacy and safety of Quxie Capsule in patients with CRC, 2 trials evaluated the efficacy and safety of Danggui Buxue decoction in CRC, and 1 trial evaluated the efficacy and safety of Gegen Qinlian decoction in postoperative CRC patients who underwent adjuvant chemotherapy. The gut microbiota regulating the function of these eight formulas is shown in **Table 2**.

**Table 2:** Gut regulating function of herbal formulas.

Formulas	Disease	Microbiota upregulating	Microbiota downregulating
Shenlingbaizhu powder (Li et al., 2020a)	Gastric cancer	*Lactobacillus*, *Bifidobacterium*, bacillus/coccus ratio	*Escherichia coli*, *Enterococcus*, *Staphylococcus*, *Peptostreptococcus*
Daikenchuto (Hanada et al., 2021)	Colon cancer	–	*Serratia* and *Bilophila* (belonging to the phylum Proteobacteria)
Danggui Buxue Decoction (Shi et al., 2021)	Colon cancer	Bacteroidetes, Epsilonbacteraeota, *Bacteroides*, norank_f_Muribaculaceae, Alloprevotella, Prevotellaceae_UCG-001, Parabacteroides	Firmicutes/Bacteroidetes, Patescribacteria, Odoribacter, and Alistipes, Lachnospiraceae_NK4A136_group, unclassified_f_Ruminococcaceae, *Lactobacillus*, unclassified_f_Lachnospiraceae, Ruminococcaceae_UCG-014
Gegen Qinlian decoction (Li et al., 2020b)	Colorectal cancer	*Bacteroides*, *Akkermansia*, *Prevotella*	*Megamonas*, *Veillonella*
Liujunzi decoction (Cheng et al. 2021a)	Advanced gastric cancer	*Escherichia coli*	*Bifidobacterium*, *Lactobacillus*, *Enterococcus faecalis*
Quxie Capsule (Sun et al., 2020)	Advanced colorectal cancer	Actinobacteria, Lachnospiraceae, Prevotella_9, Clostridia	*Bacteroides*, *Escherichia-Shigella*, *Bacteroidetes*, *Gammaproteobacteria*
Sijunzi decoction (Zhang et al., 2020)	Postoperative colorectal cancer	*Bifidobacterium*, *Lactobacillus*	–
Sijunzi decoction (Sun et al., 2012)	Postoperative colorectal cancer	*Bifidobacterium*, *Bifidobacterium*/*Escherichia coli* ratio	–

#### 3.1.2 Risk of bias in included trials

**Figure 2:**
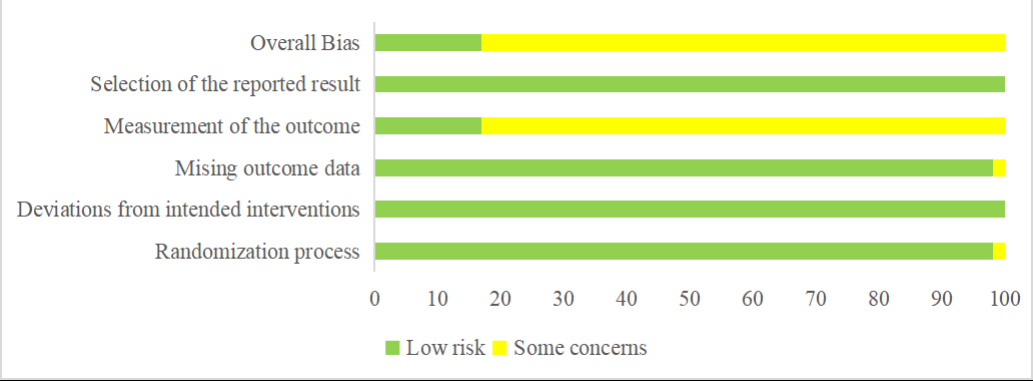
Risk of bias of included trials.

We assessed the risk of bias in 53 included trials with the RoB 2 tool. A total of 9 trials were assessed as ‘Low’ risk of bias, and 44 trials were assessed as having ‘Some concerns’. Most concerns were caused by the measurement of the outcomes since the assessment of outcomes could be influenced by knowledge of interventions that patients received. Among nine low risk-of-bias trials, five were double-blinded RCTs, two trials implemented a blind method to outcome assessors, and another two trials reported survival outcomes that may not be influenced by knowledge of interventions. The summary of the risk of bias is shown in **Figure 2**.

### 3.2 Herbal formulas with the function of gut microbiota regulation for gastric cancer

#### 3.2.1 Herbal formulas with the function of gut microbiota regulation for perioperative gastric cancer

There are nine trials that evaluated the efficacy and safety of HFGMR in perioperative patients with GC. The outcomes of gastrointestinal function recovery, which include time to bowel sound recovery, time to first flatus, and time to the first defecation, were reported in eight trials. Furthermore, five trials reported the outcome of the duration of hospitalization, and two trials reported time to first oral feeding and time to the out-of-bed activity.

##### 3.2.1.1 Gastrointestinal function recovery in perioperative gastric cancer

Three trials reported time to bowel sound recovery, and a meta-analysis of two trials showed that the herbal formulas plus enhanced recovery after surgery (ERAS) could shorten the time to bowel sound recovery by 1.63 h [MD = −1.63, 95% CI (−2.62, −0.65)] (Huang et al., 2018; Cao et al., 2020), and one trial reported that herbal formulas plus supportive care (SC) could significantly shorten the time to bowel sound recovery (Yue et al., 2019); the results are shown in **Table 3**. Eight trials reported the outcome of time to first flatus, and a meta-analysis of two trials showed that the herbal formulas plus enteral nutrition (EN) could shorten the time to first flatus by 9.69 h [MD = −9.69, 95% CI (−10.89, −8.48)] (Li et al., 2020; Yu and Ren, 2019; Li et al., 2020), three trials reported that the herbal formulas plus ERAS could significantly shorten time to first flatus (Huang et al., 2018; Chen et al., 2020; Cao et al., 2020), two trials reported that the herbal formulas plus SC could significantly shorten time to first flatus (Fu et al., 2007; Yue et al., 2019), and another trial reported that no significant difference was observed between the herbal formulas plus SC and SC groups (Yoshikawa et al., 2015), but a meta-analysis was not conducted due to the existence of considerable heterogeneity within these two subgroups; the results are shown in **Table 3**. Six trials reported the outcome of time to the first defecation, one trial reported that herbal formulas plus ERAS could shorten the time to first defecation (Chen et al., 2020), and a meta-analysis of two trials showed that there was no significant difference in time to the first defecation between patients who received herbal formulas plus EN and the EN group [MD = −0.72, 95% CI (−1.68, 0.25)] (Li et al., 2020; Yu and Ren, 2019), two trials reported that the herbal formulas plus SC could significantly shorten time to first defecation (Yoshikawa et al., 2015; Yue et al., 2019), and another trial reported that no significant difference was observed between the herbal formulas plus SC and SC groups (Akamaru et al., 2015). A meta-analysis was not conducted due to the existence of considerable heterogeneity within the subgroup; the results are shown in **Table 3**.

**Table 3:** Results of efficacy and safety of herbal formulas with function of gut microbiota regulation in GC and CRC.

Outcomes/subgroups	Number of trials	Number of participants	Effect estimate	I^2^
Perioperative GC				
1. Time to bowel sound recovery				
Herbal formulas + ERAS *vs*. ERAS	2	120	MD = −1.63, 95% CI (−2.62, −0.65)	0
Herbal formulas + SC *vs*. SC	1	80	MD = −22.36, 95% CI (−25.05, −19.67)	–
2. Time to first flatus				
Herbal formulas + ERAS *vs*. ERAS	3	204	–	84.00%
Herbal formulas + SC *vs*. SC	3	355	–	99.02%
Herbal formulas + EN *vs*. EN	2	200	MD = −9.69, 95% CI (−10.89, −8.48)	0
3. Time to first defecation				
Herbal formulas + ERAS *vs*. ERAS	1	84	MD = −6.65, 95% CI (−8.88, −4.42)	–
Herbal formulas + SC *vs*. SC	3	356	–	93.29%
Herbal formulas + EN *vs*. EN	2	200	MD = −0.72, 95% CI (−1.68, 0.25)	0
4. Time to first oral feeding				
Herbal formulas + EN *vs*. EN	2	200	MD = −2.74, 95% CI (−3.94, −1.54)	0
5. Time to out-of-bed activity				
Herbal formulas + EN *vs*. EN	2	200	MD = −2.11, 95% CI (−3.04, −1.19)	0
6. Duration of hospitalization				
Herbal formulas + ERAS *vs*. ERAS	2	144	MD = −2.09, 95% CI (−2.75, −1.43)	0
Herbal formulas + EN *vs*. EN	2	200	MD = −4.00, 95% CI (−5.16, −2.84)	0
7. KPS score				
Herbal formulas + ERAS *vs*. ERAS	1	60	MD = 4.58, 95% CI (3.53, 5.63)	–
8. Safety outcomes				
8.1 Intestinal obstruction				
Herbal formulas + SC *vs*. SC	2	277	RR = 1.02, 95% CI (0.25, 4.21)	0
8.2 Diarrhea				
Herbal formulas + SC *vs*. SC	1	195	RR = 2.06, 95% CI (0.19, 22.37)	–
8.3 Ventosity				
Herbal formulas + SC *vs*. SC	1	80	RR = 0.25, 95% CI (0.03, 2.14)	–
8.4 Nausea and vomiting				
Herbal formulas + SC *vs*. SC	2	275	RR = 0.80, 95% CI (0.23, 2.86)	0
Postoperative GC				
1. 2-year survival rate				
Herbal formulas + FOLFOX4 *vs*. FOLFOX4	1	76	RR = 1.03, 95% CI (0.91, 1.16)	–
2. QoL				
Herbal formulas + FOLFOX4 *vs*. FOLFOX4	1	112	MD = 0.94, 95% CI (0.70, 1.18)	
3. KPS score				
Herbal formulas + FOLFOX4 *vs*. FOLFOX4	1	76	MD = 0.94, 95% CI (7.55, 11.03)	
4. Safety outcomes				
4.1 Leucopenia				
Herbal formulas + FOLFOX4 *vs*. FOLFOX4	1	112	RR = 0.78, 95% CI (0.50, 1.20)	–
4.2 Anemia				
Herbal formulas + FOLFOX4 *vs*. FOLFOX4	2	188	RR = 0.66, 95% CI (0.33, 1.31)	60.27%
4.3 Thrombocytopenia				
Herbal formulas + FOLFOX4 *vs*. FOLFOX4	2	188	RR = 0.54, 95% CI (0.16, 1.82)	80.29%
4.4 Diarrhea				
Herbal formulas + FOLFOX4 *vs*. FOLFOX4	1	112	RR = 0.50, 95% CI (0.26, 0.97)	–
4.5 Nausea and vomiting				
Herbal formulas + FOLFOX4 *vs*. FOLFOX4	2	188	RR = 0.49, 95% CI (0.30, 0.80)	0
4.6 Anorexia				
Herbal formulas + FOLFOX4 *vs*. FOLFOX4	1	112	RR = 0.61, 95% CI (0.44, 0.85)	–
4.7 Hepatic and renal dysfunction				
Herbal formulas + FOLFOX4 *vs*. FOLFOX4	1	112	RR = 0.75, 95% CI (0.28, 2.02)	–
4.8 Peripheral neurotoxicity				
Herbal formulas + FOLFOX4 *vs*. FOLFOX4	2	188	RR = 0.46, 95% CI (0.22, 0.97)	12.32%
Advanced GC				
1. mPFS				
Herbal formulas + ECF chemotherapy *vs*. ECF chemotherapy	1	68	MD = 0.34, 95% CI (0.20, 0.48)	–
2. mOS				
Herbal formulas + ECF chemotherapy *vs*. ECF chemotherapy	1	68	MD = 0.24, 95% CI (0.13, 0.35)	–
3. ORR				
Herbal formulas + FOLFOX *vs*. FOLFOX	2	192	RR = 1.67, 95% CI (1.21, 2.30)	0
Herbal formulas + 5-FU+CF+paclitaxel *vs*. 5-FU+CF+paclitaxel	2	151	RR = 1.03, 95% CI (0.79, 1.33)	0
Herbal formulas + other chemotherapy *vs*. other chemotherapy	3	330	RR = 1.40, 95% CI (1.18, 1.66)	0
Herbal formulas + apatinib *vs*. apatinib	1	60	RR = 1.89, 95% CI (1.01, 3.55)	–
4. DCR				
Herbal formulas + FOLFOX *vs*. FOLFOX	2	192	RR = 1.32, 95% CI (1.11, 1.58)	0
Herbal formulas + 5-FU+CF+paclitaxel *vs*. 5-FU+CF+paclitaxel	2	151	RR = 0.96, 95% CI (0.82, 1.13)	0
Herbal formulas + other chemotherapy *vs*. other chemotherapy	3	330	RR = 1.10, 95% CI (1.02, 1.18)	0
Herbal formula + apatinib *vs*. apatinib	1	60	RR = 1.40, 95% CI (1.07, 1.83)	–
5. QoL score				
Herbal formula + TXELOX regimen *vs*. TXELOX regimen	1	78	MD = 3.62, 95% CI (3.31, 3.93)	–
6. Performance status				
6.1 KPS score				
Herbal formulas + chemotherapy/apatinib *vs*. chemotherapy/apatinib	6	442	–	99.89%
6.2 KPS improvement rate				
Herbal formulas + 5-FU+CF+paclitaxel *vs*. 5-FU+CF+paclitaxel	2	129	RR = 1.56, 95% CI (1.17, 2.09)	0
Herbal formula + TS chemotherapy *vs*. TS chemotherapy	1	100	RR = 1.39, 95% CI (0.88, 2.20)	–
7. Safety outcomes				
7.1 Leucopenia				
Herbal formulas + chemotherapy *vs*. chemotherapy	7	619	RR = 0.83, 95% CI (0.70, 0.98)	0
Herbal formulas + apatinib *vs*. apatinib	1	60	RR = 0.29, 95% CI (0.12, 0.69)	–
7.2 Neutropenia				
Herbal formulas + chemotherapy *vs*. chemotherapy	2	128	RR = 0.73, 95% CI (0.56, 0.95)	0
Herbal formulas + apatinib *vs*. apatinib	1	60	RR = 0.30, 95% CI (0.09, 0.98)	–
7.3 Thrombocytopenia				
Herbal formulas + chemotherapy *vs*. chemotherapy	3	241	RR = 0.82, 95% CI (0.48, 1.39)	0
Herbal formulas + apatinib *vs*. apatinib	1	60	RR = 0.25, 95% CI (0.06, 1.08)	–
7.4 Anemia				
Herbal formulas + chemotherapy *vs*. chemotherapy	2	129	RR = 0.56, 95% CI (0.35, 0.89)	12.65%
Herbal formulas + apatinib *vs*. apatinib	1	60	RR = 0.25, 95% CI (0.08, 0.80)	–
7.5 Nausea and vomiting				
Herbal formulas + chemotherapy *vs*. chemotherapy	8	693	RR = 0.62, 95% CI (0.45, 0.85)	54.57%
7.6 Diarrhea				
Herbal formulas + chemotherapy *vs*. chemotherapy	4	379	RR = 0.70, 95% CI (0.53, 0.91)	18.85%
Herbal formulas + apatinib *vs*. apatinib	1	60	RR = 0.22, 95% CI (0.05, 0.94)	–
7.7 Anorexia				
Herbal formulas + chemotherapy *vs*. chemotherapy	4	326	RR = 0.73, 95% CI (0.51, 1.06)	54.69%
Herbal formulas + apatinib *vs*. apatinib	1	60	RR = 0.11, 95% CI (0.01, 0.82)	–
7.8 Hepatic dysfunction				
Herbal formulas + chemotherapy *vs*. chemotherapy	3	280	RR = 0.63, 95% CI (0.18, 2.17)	37.18%
Herbal formulas + apatinib *vs*. apatinib	1	60	RR = 0.25, 95% CI (0.06, 1.08)	–
7.9 Fatigue				
Herbal formulas + chemotherapy *vs*. chemotherapy	1	82	RR = 0.49, 95% CI (0.31, 0.77)	–
Herbal formulas + apatinib *vs*. apatinib	1	60	RR = 0.13, 95% CI (0.02, 0.94)	–
Perioperative CRC				
1. Time to bowel sound recovery				
Herbal formulas + EN *vs*. EN	1	110	MD = −4.74, 95% CI (−6.08, −3.40)	–
2. Time to first flatus				
Herbal formulas + ERAS *vs*. ERAS	1	69	MD = −10.61, 95% CI (−16.77, −4.45)	–
Herbal formulas + EN *vs*. EN	2	170	–	96.15%
Herbal formulas + SC *vs*. SC	3	153	MD = −3.26, 95% CI (−13.75, 7.23)	69.26%
3. Time to first defecation				
Herbal formulas + ERAS *vs*. ERAS	1	69	MD = −12.12, 95% CI (−17.16, −7.08)	–
Herbal formulas + EN *vs*. EN	2	170	–	97.57%
Herbal formulas + SC *vs*. SC	2	82	–	95.40%
4. Duration of hospitalization				
Herbal formulas + ERAS *vs*. ERAS	1	69	MD = −2.29, 95% CI (−3.16, −1.42)	–
Herbal formulas + EN *vs*. EN	1	60	MD = −1.50, 95% CI (−3.29, 0.29)	–
Herbal formulas + SC *vs*. SC	1	51	MD = −0.46, 95% CI (−1.18, 0.26)	–
5. Time to first oral feeding				
Herbal formulas + SC *vs*. SC	1	51	MD = −6.45, 95% CI (−14.23, 1.33)	–
6. Safety outcomes				
6.1 Nausea and vomiting				
Herbal formulas + EN *vs*. EN	1	110	RR = 2.00, 95% CI (0.19, 21.42)	–
6.2 Diarrhea				
Herbal formulas + EN *vs*. EN	1	110	RR = 1.00, 95% CI (0.06, 15.59)	–
Herbal formulas + SC *vs*. SC	2	102	RR = 0.70, 95% CI (0.04, 11.33)	39.55%
6.3 Fever				
Herbal formulas + EN *vs*. EN	1	110	RR = 0.33, 95% CI (0.01, 8.01)	–
6.4 Erythema				
Herbal formulas + EN *vs*. EN	1	110	RR = 0.33, 95% CI (0.01, 8.01)	–
Postoperative CRC				
1. Mean PFS				
Quxie Capsule *vs*. control	2	140	MD = 8.70, 95% CI (3.27, 14.13)	64.42%
2. KPS score				
Herbal formulas + chemotherapy *vs*. chemotherapy	8	682	–	99.95%
3. KPS improvement rate				
Herbal formulas + chemotherapy *vs*. chemotherapy	4	416	RR = 1.96, 95% CI (1.38, 2.79)	0.00%
4. Safety outcomes				
4.1 Leucopenia				
Herbal formulas + chemotherapy *vs*. chemotherapy	6	487	RR = 0.83, 95% CI (0.71, 0.98)	27.72%
4.2 Neutropenia				
Herbal formulas + chemotherapy *vs*. chemotherapy	2	149	RR = 0.92, 95% CI (0.82, 1.02)	0.01%
4.3 Anemia				
Herbal formulas + chemotherapy *vs*. chemotherapy	4	316	RR = 1.00, 95% CI (0.91, 1.11)	0
4.4 Thrombocytopenia				
Herbal formulas + chemotherapy *vs*. chemotherapy	4	308	RR = 0.78, 95% CI (0.55, 1.09)	0
4.5 Nausea and vomiting				
Herbal formulas + chemotherapy *vs*. chemotherapy	6	487	RR = 0.68, 95% CI (0.50, 0.92)	54.84%
4.6 Diarrhea				
Herbal formulas + chemotherapy *vs*. chemotherapy	3	220	RR = 0.53, 95% CI (0.22, 1.29)	81.41%
4.7 Peripheral neurotoxicity				
Herbal formulas + chemotherapy *vs*. chemotherapy	3	226	RR = 0.84, 95% CI (0.61, 1.17)	0
Advanced CRC				
1. 1-year survival rate				
Quxie Capsule + standard treatment *vs*. standard treatment	1	54	RR = 1.55, 95% CI (1.15, 2.08)	–
2. 2-year survival rate				
Quxie Capsule + standard treatment *vs*. standard treatment	1	54	RR = 3.02, 95% CI (1.25, 7.28)	–
3. 3-year survival rate				
Quxie Capsule + standard treatment *vs*. standard treatment	1	54	RR = 1.16, 95% CI (0.08, 17.60)	–
4. mOS				
Quxie Capsule + standard treatment *vs*. standard treatment	2	91	–	82.94%
5. ORR				
Herbal formulas + FOLFOX *vs*. FOLFOX	3	306	RR = 1.44, 95% CI (0.92, 2.25)	61.24%
Herbal formulas + RTX-based chemotherapy *vs*. RTX-based chemotherapy	2	105	RR = 1.02, 95% CI (0.57, 1.81)	0
6. DCR				
Herbal formulas + FOLFOX *vs*. FOLFOX	3	306	–	83.88%
Herbal formulas + RTX-based chemotherapy *vs*. RTX-based chemotherapy	2	105	RR = 1.09, 95% CI (0.89, 1.34)	0
7. QoL score				
Herbal formulas + chemotherapy *vs*. chemotherapy	2	157	–	96.02%
8. QLQ-C30 score				
Herbal formulas + chemotherapy *vs*. chemotherapy	1	106	MD = 6.93, 95% CI (6.87, 6.99)	–
9. KPS score				
Herbal formulas + chemotherapy *vs*. chemotherapy	4	273	–	99.22%
10. KPS improvement rate				
Herbal formulas + chemotherapy *vs*. chemotherapy	3	187	RR = 1.62, 95% CI (1.13, 2.32)	0
11. Safety outcomes				
11.1 Myelosuppression				
Herbal formulas + chemotherapy *vs*. chemotherapy	4	295	RR = 0.58, 95% CI (0.42, 0.79)	0
11.2 Nausea and vomiting				
Herbal formulas + chemotherapy *vs*. chemotherapy	5	373	RR = 0.67, 95% CI (0.50, 0.91)	0
11.3 Diarrhea				
Herbal formulas + chemotherapy *vs*. chemotherapy	5	308	RR = 0.41, 95% CI (0.25, 0.67)	0
11.4 Hepatic and renal dysfunction				
Herbal formulas + chemotherapy *vs*. chemotherapy	5	334	RR = 0.51, 95% CI (0.33, 0.79)	0

GC, gastric cancer; CRC, colorectal cancer; ERAS, enhanced recovery after surgery; SC, supportive care; EN, enteral nutrition; KPS, Karnofsky performance status; QoL, quality of life; mPFS, median progression-free survival; mOS, median overall survival; ORR, objective response rate; DCR, disease control rate; PFS, progression-free survival; RR, risk ratio.

##### 3.2.1.2 Other outcomes in perioperative gastric cancer

**Figure 3:**
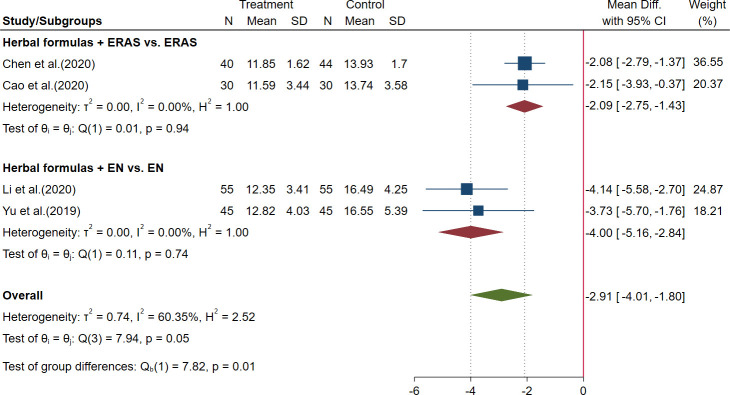
Forest plot of duration of hospitalization in perioperative gastric cancer (GC).

Five trials reported the duration of hospitalization: four of these trials reported that HFGMR could shorten the duration of hospitalization, while one trial reported there was no significant difference between the two groups in postoperative hospital stay and did not provide the data in detail (Yoshikawa et al., 2015). The meta-analysis showed that the herbal medicine plus ERAS could shorten the duration of hospitalization by 2.09 days [MD = −2.09, 95% CI (−2.75, −1.43)] (Chen et al., 2020; Cao et al., 2020) and could shorten the duration of hospitalization by 4.00 days when combined with EN [MD = −4.00, 95% CI (−5.16, −2.84)] (Li, 2020; Yu and Ren, 2019). The results are shown in **Figure 3** and **Table 3**.

The meta-analysis of two trials showed that herbal medicine plus EN could shorten the time to first oral feeding by 2.74 h [MD = −2.74, 95% CI (−3.94, −1.54)] and shorten the time to out-of-bed activity by 2.11 h [MD = −2.11, 95% CI (−3.04, −1.19)] (Li, 2020; Yu and Ren, 2019). The results are shown in **Table 3**. Two trials reported that there was no significant difference in QOL scores between the treatment groups of herbal medicine and the control groups (Yoshikawa et al., 2015; Akamaru et al., 2015). One trial reported that herbal formula could help improve the Karnofsky performance status (KPS) score significantly (Cao et al., 2020); the result is shown in **Table 3**.

##### 3.2.1.3 Safety outcomes in perioperative gastric cancer

**Figure 4:**
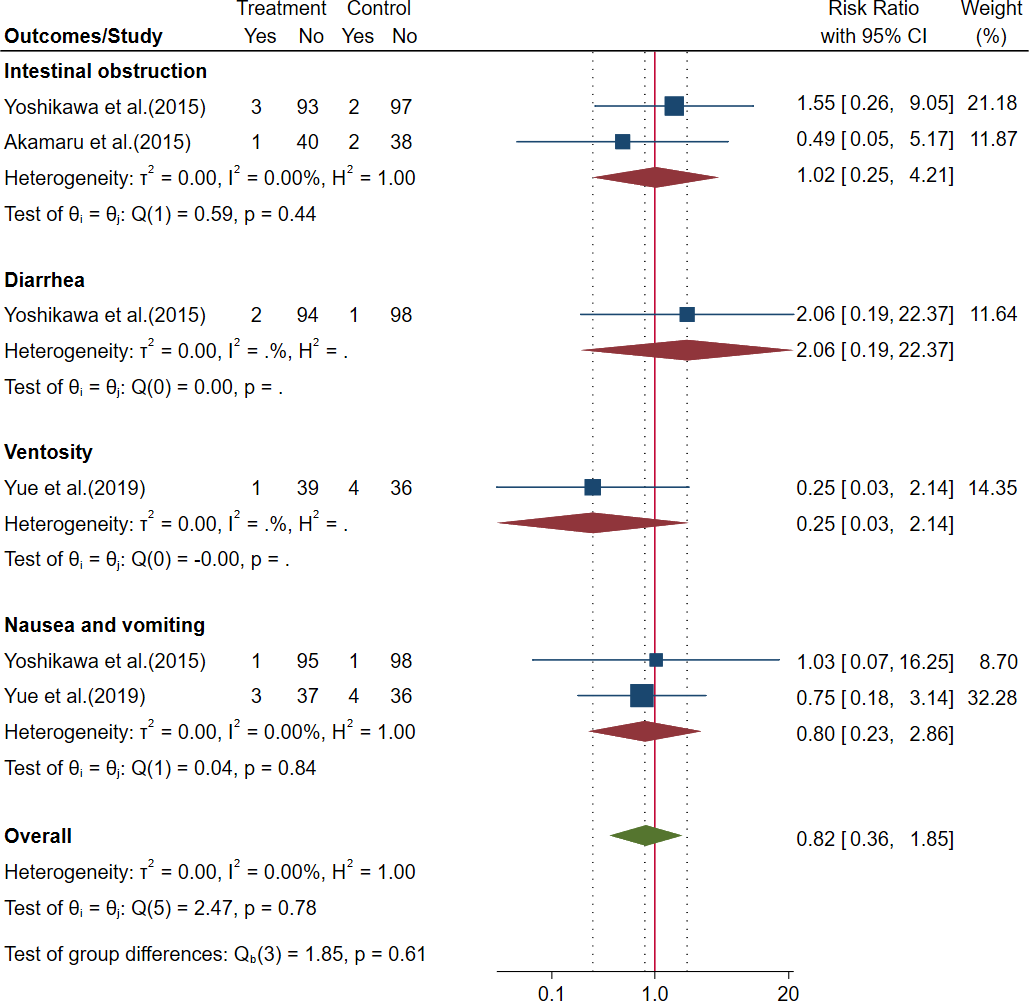
Forest plot of adverse events (AEs) in perioperative gastric cancer (GC).

There were no significant differences in the incidence of intestinal obstruction, diarrhea, ventosity, nausea, and vomiting; the results are shown in **Figure 4** and **Table 3**.

#### 3.2.2 Herbal formulas with the function of gut microbiota regulation plus adjuvant chemotherapy for postoperative gastric cancer

Two trials evaluated the clinical efficacy and safety of HFGMR plus FOLFOX4 adjuvant chemotherapy compared to FOLFOX4 chemotherapy alone for postoperative patients with GC (Li, 2017; Li et al., 2020a).

##### 3.2.2.1 Efficacy outcomes in postoperative gastric cancer

One trial reported long-term survival outcomes of herbal formulas plus FOLFOX4 chemotherapy, and there was no significant difference in the 2-year survival rate between herbal formulas plus chemotherapy and chemotherapy alone (94.74% *vs*. 92.11%) (Li, 2017). One trial reported a QoL score, and the result demonstrated that herbal formulas plus chemotherapy could improve the QoL score by 0.94 points [MD = 0.94, 95% CI (0.70, 1.18)] (Li et al., 2020a). Another trial showed that herbal formula plus chemotherapy could improve the KPS score by 9.29 points [MD = 0.94, 95% CI (7.55, 11.03)], compared to FOLFOX4 chemotherapy alone (Li, 2017).

##### 3.2.2.2 Safety outcomes in postoperative GC

**Figure 5:**
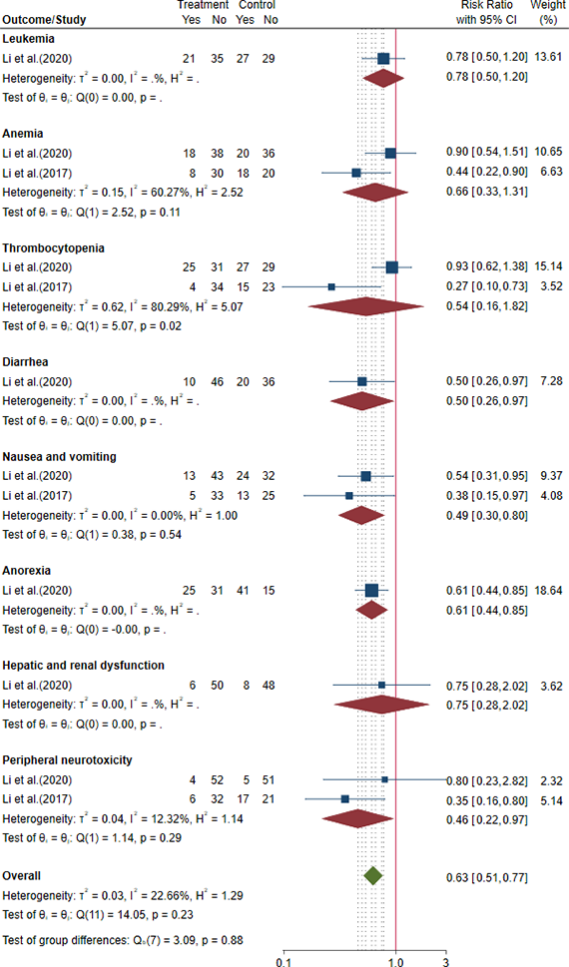
Forest plot of adverse events (AEs) in postoperative gastric cancer (GC).

Both two trials reported AEs (Li, 2017; Li et al., 2020a). The results showed that compared to FOLFOX chemotherapy alone, HFGMR plus FOLFOX chemotherapy may decrease the incidence of diarrhea, nausea and vomiting, anorexia, and peripheral neurotoxicity, and there were no significant differences in the incidence of leucopenia, anemia, thrombocytopenia, and hepatic and renal dysfunction. The results are shown in **Figure 5** and **Table 3**.

#### 3.2.3 Herbal formulas with the function of gut microbiota regulation for advanced gastric cancer

Twelve trials evaluated the efficacy and safety of HFGMR in patients with advanced GC. Eleven trials compared herbal formulas plus chemotherapy with chemotherapy alone, and another trial compared herbal formulas plus apatinib with apatinib alone (Wang et al., 2019).

##### 3.2.3.1 Long-term survival outcomes in advanced gastric cancer

One trial reported the long-term survival outcomes of median progression-free survival (mPFS) and median overall survival (mOS); the results showed that compared to ECF (Epirubicin + Cisplatin + Fluorouracil) regimen chemotherapy alone, herbal formula plus ECF regimen chemotherapy could significantly prolong the mPFS (5.86 ± 0.26 *vs*. 5.52 ± 0.33 months) and mOS (13.08 ± 0.25 *vs*. 12.84 ± 0.19 months) (Shen et al., 2021).

##### 3.2.3.2 Outcomes of tumor response rate in advanced gastric cancer

**Figure 6:**
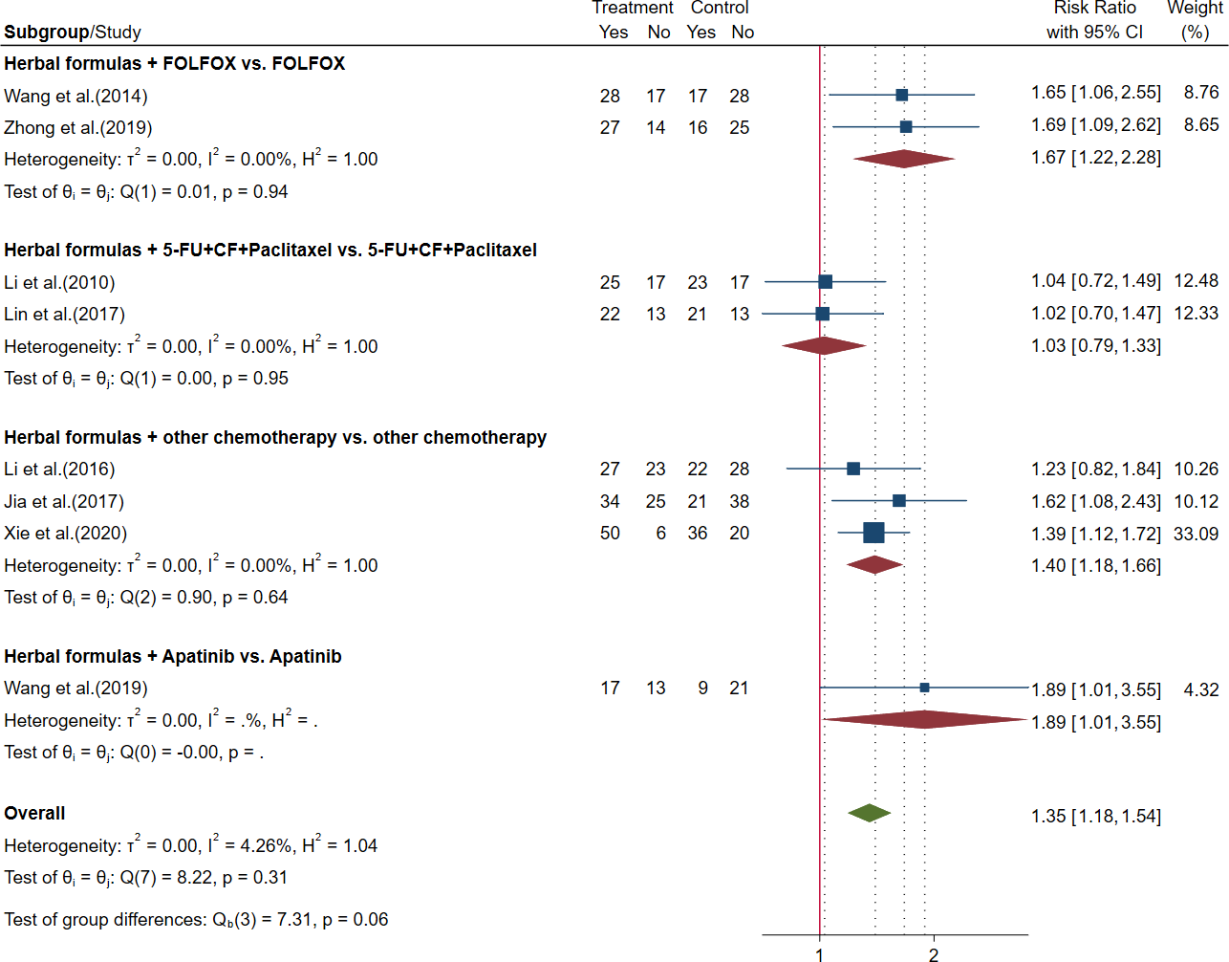
Forest plot of objective response rate (ORR) in advanced gastric cancer (GC).

**Figure 7:**
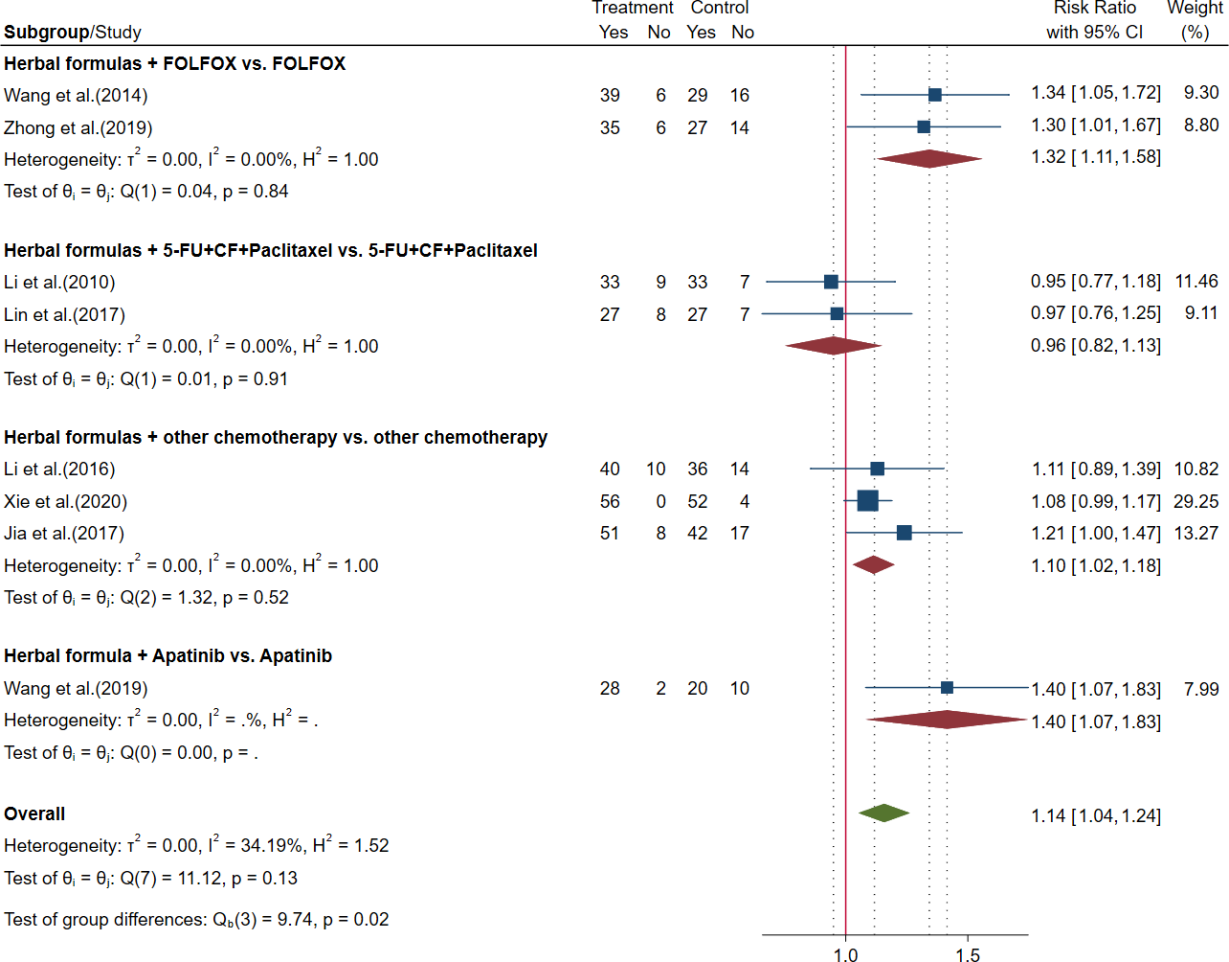
Forest plot of disease control rate (DCR) in advanced gastric cancer (GC).

Eight trials reported the outcomes of ORR and disease control rate (DCR). The meta-analysis showed that HFGMR plus FOLFOX regimen chemotherapy and other regimen chemotherapy could significantly increase the ORR and DCR, but no significant differences were observed in ORR and DCR between herbal formulas plus 5-FU+CF+paclitaxel chemotherapy and 5-FU+CF+paclitaxel regimen alone. The results are shown in **Figures 6**, **7** and **Table 3**.

##### 3.2.3.3 Other efficacy outcomes in advanced gastric cancer

One trial demonstrated that compared to TXELOX (Taxol + Xeloda + Oxaliplatin) regimen chemotherapy alone, herbal formulas plus TXELOX may improve QoL score by 3.62 points [MD = 3.62, 95% CI (3.31, 3.93)] (Wang and Yan, 2020). Five trials reported that herbal formulas could help to improve the KPS score, but the meta-analysis was not conducted owing to the existence of considerable heterogeneity (Zhang and Su, 2008; Wang et al., 2014; Zhong et al., 2019; Wang and Yan, 2020; Wang et al., 2019). As for KPS improvement rate, a meta-analysis of two trials showed that compared to 5-FU+CF+paclitaxel regimen chemotherapy alone, herbal formulas plus 5-FU+CF+paclitaxel could increase the improvement rate by 56% [RR = 1.56, 95% CI (1.17, 2.09)] (Lin et al., 2017; Lai et al., 2018), and another trial showed that the KPS improvement rate may not differ between herbal formulas plus TS regimen chemotherapy and TS regimen alone (Li et al., 2016).

##### 3.2.3.4 Safety outcomes in advanced gastric cancer

Eleven trials reported the incidence of AEs in advanced GC. The meta-analysis showed that compared to chemotherapy alone, herbal formulas plus chemotherapy could decrease the incidence of leucopenia by 17% [RR = 0.83, 95% CI (0.70, 0.98)], neutropenia by 27% [RR = 0.73, 95% CI (0.56, 0.95)], anemia by 46% [RR = 0.56, 95% CI (0.35, 0.89)], nausea and vomiting by 38% [RR = 0.62, 95% CI (0.45, 0.85)], diarrhea by 30% [RR = 0.70, 95% CI (0.53, 0.91)], and fatigue by 51% [RR = 0.49, 95% CI (0.31, 0.77)], and there were no significant differences in the incidence of thrombocytopenia, anorexia, and hepatic dysfunction; the details of the results are shown in **Table 3**.

### 3.3 Herbal formulas with the function of gut microbiota regulation for colorectal cancer

#### 3.3.1 Herbal formulas with the function of gut microbiota regulation for perioperative colorectal cancer

There are eight trials that evaluated the efficacy and safety of HFGMR in perioperative patients with CRC (Chen, 2013; Yaegashi et al., 2014; Katsuno et al., 2015; Katsuno et al., 2016; Hanada et al., 2021; Yang et al., 2021; Wakasugi et al., 2020; Zhang, 2020). The outcomes of gastrointestinal function recovery, which include time to bowel sound recovery, time to first flatus, and time to the first defecation were reported in six trials (Chen, 2013; Yaegashi et al., 2014; Katsuno et al., 2016; Zhang, 2020; Wakasugi et al., 2020; Yang et al., 2021). Moreover, duration of hospitalization, time to first oral feeding, time to an out-of-bed activity, and safety outcomes were also reported in these trials.

##### 3.3.1.1 Gastrointestinal function recovery in perioperative colorectal cancer

One trial reported that compared to EN alone, herbal formula plus EN may shorten the time to bowel sound recovery by 4.74 h (Yang et al., 2021). Six trials reported the outcome of time to first flatus, one trial showed that compared to ERAS alone, herbal formula plus ERAS may shorten the time to first flatus by 10.61 h (Zhang, 2020), and two trials showed that compared to ERAS alone, herbal formula plus ERAS may shorten the time to first flatus by 10.61 h; owing to the considerable statistic heterogeneity between two trials, a meta-analysis was not conducted (Chen, 2013; Yang et al., 2021). The meta-analysis of three trials demonstrated that there was no significant difference in time to first flatus between the patients who received Daikenchuto plus SC and SC [MD = −3.26, 95% CI (−13.75, 7.23)] (Yaegashi et al., 2014; Katsuno et al., 2016; Wakasugi et al., 2020). Five trials reported the outcome of time to the first defecation, four trials demonstrated that herbal formulas may shorten the time to first defecation (Chen, 2013; Yaegashi et al., 2014; Zhang, 2020; Yang et al., 2021), and another trial concluded that herbal formula Daikenchuto may potentially inhibit diarrhea and prolong the time to first defecation (Wakasugi et al., 2020). The results are shown in **Table 3**.

##### 3.3.1.2 Other efficacy outcomes in perioperative colorectal cancer

Three trials reported the duration of hospitalization; one trial showed that compared to ERAS alone, herbal formula Liujunzi decoction may shorten the duration of hospitalization by 2.29 days (Zhang, 2020); another two trials showed that herbal formula combined with EN or SC may shorten the duration of hospitalization, but no significant differences were observed (Chen, 2013; Yaegashi et al., 2014). One trial demonstrated that there was no significant difference in time to oral feeding between patients who received Daikenchuto plus SC and SC alone (Yaegashi et al., 2014).

##### 3.3.1.3 Safety outcomes in perioperative colorectal cancer

Three trials reported the incidence of AEs, but no significant differences in the incidence of nausea and vomiting, diarrhea, fever, or erythema were observed between the two groups (Yaegashi et al., 2014; Katsuno et al., 2016; Yang et al., 2021). The details are shown in **Table 3**.

#### 3.3.2 Herbal formulas with the function of gut microbiota regulation for postoperative colorectal cancer

Ten trials evaluated the efficacy of HFGMR plus adjuvant chemotherapy in postoperative patients with CRC, patients in eight trials received FOLFOX regimen chemotherapy (Bai et al., 2017, Wang and Liu, 2020, Yue, 2016; Wang et al., 2018; Dong, 2018; Liu et al., 2019; Liu and Xia, 2019; Sun, 2020), and patients of two trials received multiple regimens, which included FOLFOX, FOLFIRI, and XELOX (Lim, 2014; Tong et al., 2017). Two trials evaluated the long-term efficacy of the herbal drug Quxie Capsule after the patients finished adjuvant chemotherapy (Yang et al., 2007; Yang, 2015).

##### 3.3.2.1 Long-term efficacy outcomes for postoperative colorectal cancer

Two trials reported the long-term efficacy outcome of mean PFS median progression-free survival (Yang et al., 2007; Yang, 2015). The meta-analysis of these two trials showed that the intervention of Quxie Capsule after adjuvant chemotherapy may prolong the mean PFS by 8.70 months [MD = 8.70, 95% CI (3.27, 14.13)].

##### 3.3.2.2 Other efficacy outcomes for postoperative colorectal cancer

**Figure 8:**
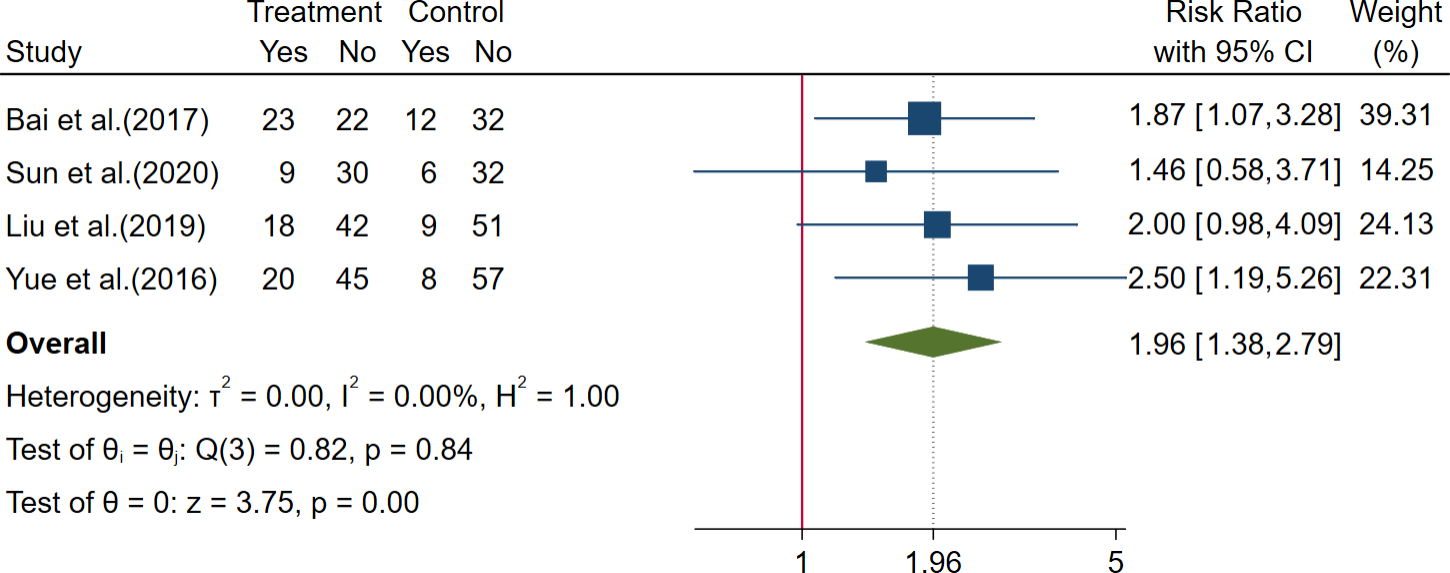
Forest plot of Karnofsky performance status (KPS) improvement rate in postoperative colorectal cancer (CRC).

Two trials reported QoL outcomes of EQRTC QLQ-C30 score; these two trials showed that modified Liujunzi decoction may help to improve the body function, character function, and emotion function and help to improve the symptom of fatigue, nausea and vomiting, diarrhea, and anorexia (Tong et al., 2017; Liu and Xia, 2019). Eight trials reported the KPS score, and seven of these trials showed that herbal formulas plus chemotherapy may increase the KPS score (Wang and Liu, 2020, Yang, 2015; Yue, 2016; Dong, 2018; Wang et al., 2018; Liu et al., 2019; Liu and Xia, 2019), while another one showed that there was no significant difference in KPS score between two groups (Lim, 2014); a meta-analysis was not performed for this outcome owing to the existence of considerable heterogeneity. Four trials reported the KPS improvement rate, a meta-analysis showed that compared to chemotherapy alone, herbal formulas plus chemotherapy could increase the KPS improvement rate by 96% [RR = 1.96, 95% CI (1.38, 2.79)] (Yue, 2016; Bai et al., 2017; Liu et al., 2019; Sun, 2020); the result is shown in **Figure 8** and **Table 3**.

##### 3.3.2.3 Safety outcomes for postoperative colorectal cancer

Five trials reported the AEs in postoperative patients with CRC, a meta-analysis showed that compared to chemotherapy alone, herbal formulas plus chemotherapy may decrease the incidence of leucopenia and nausea and vomiting, and there were no significant differences in the incidence of neutropenia, anemia, thrombocytopenia, diarrhea, and peripheral neurotoxicity (Bai et al., 2017, Wang and Liu, 2020, Lim, 2014; Dong, 2018; Sun, 2020); the results are shown in **Table 3**.

#### 3.3.3 Herbal formulas with the function of gut microbiota regulation for advanced colorectal cancer

##### 3.3.3.1 Long-term efficacy outcomes for advanced colorectal cancer

Two trials reported long-term survival outcomes (Yang et al., 2008, Zhang et al., 2021a. One trial showed that the 1-year survival rate and 2-year survival rate in patients who received Quxie Capsule were higher than that in patients who received standard treatment by 55% [RR = 1.55, 95% CI (1.15, 2.08)] and 202% [RR = 3.02, 95% CI (1.25, 7.28)], and no significant difference was observed in 3-year survival rate between two groups (Zhang et al., 2021a). Both two trials reported that herbal drugs Quxie Capsule may prolong the mOS in advanced CRC, but a meta-analysis was not performed owing to the existence of considerable heterogeneity (Yang et al., 2008, Zhang et al., 2021a). The results of survival outcomes are shown in **Table 3**.

##### 3.3.3.2 Outcomes of tumor response rate for advanced colorectal cancer

**Figure 9:**
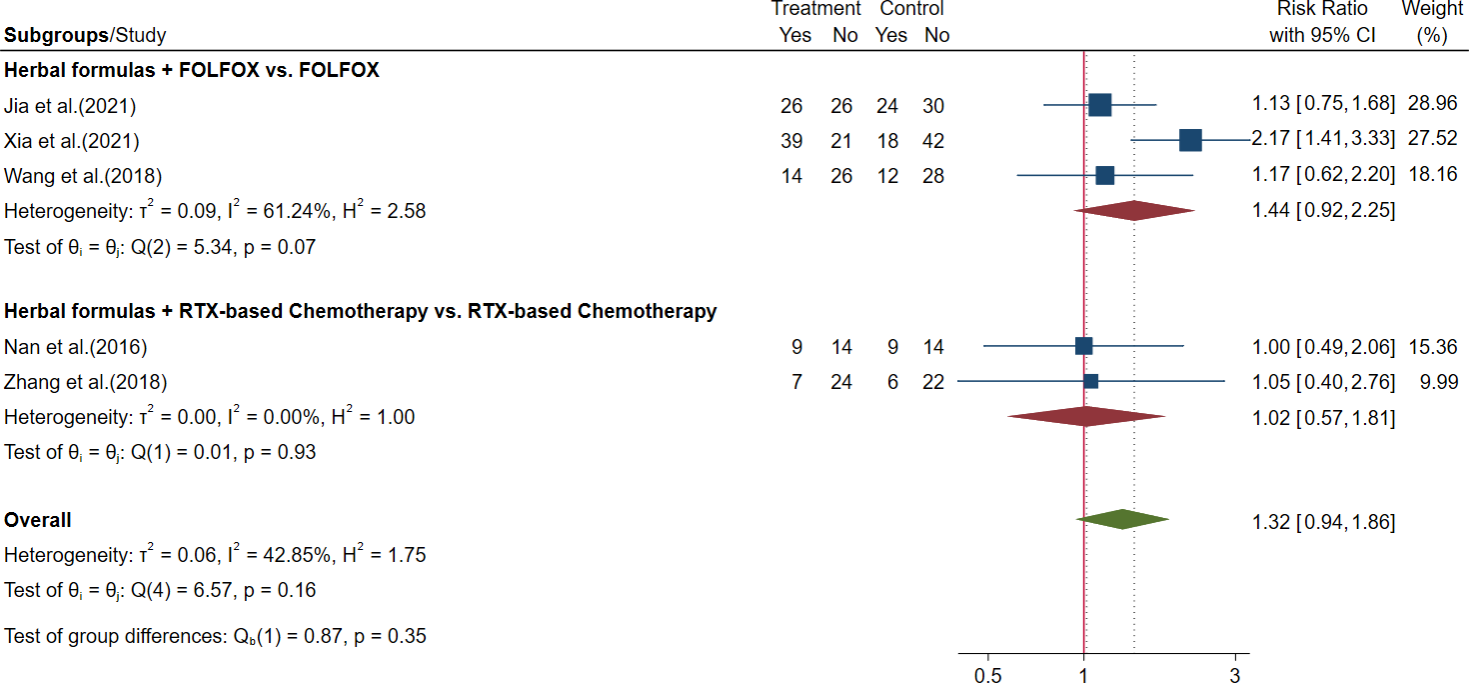
Forest plot of objective response rate (ORR) in advanced colorectal cancer (CRC).

**Figure 10:**
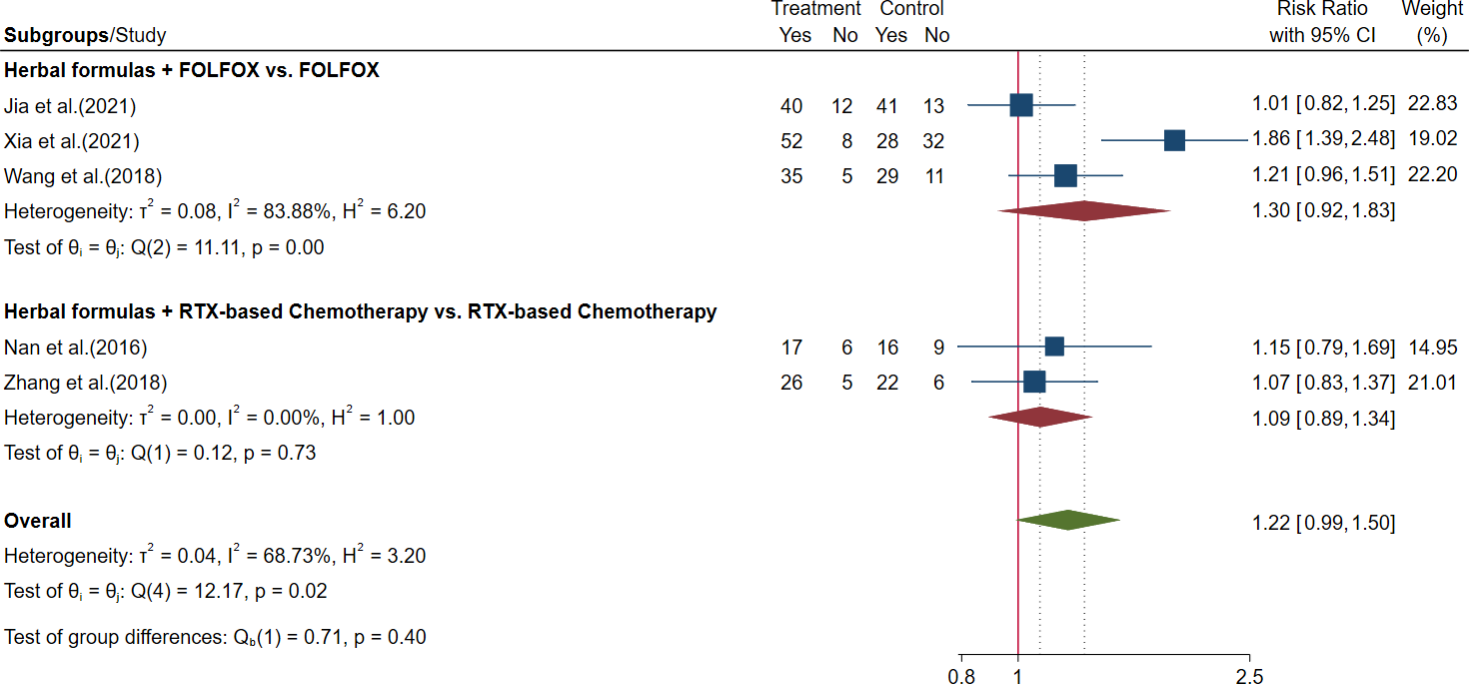
Forest plot of disease control rate (DCR) in advanced colorectal cancer (CRC).

Five trials evaluated the TRR of herbal formulas plus chemotherapy, and outcomes of ORR and DCR were reported (Nan and Li, 2016, Zhang et al., 2018, Jia et al., 2021, Jia and Dong, 2019; Wang et al., 2018; Xia et al., 2021). The meta-analysis showed that there were no significant differences in ORR or DCR between patients who received herbal formulas plus chemotherapy and patients who received chemotherapy alone. The results are shown in **Figures 9**, **10** and **Table 3**.

##### 3.3.3.3 Other efficacy outcomes for advanced colorectal cancer

Two trials demonstrated that compared to chemotherapy alone, herbal formulas plus chemotherapy may increase the QoL score (Yang et al., 2008; Xia et al., 2021). Four trials reported the outcome of KPS score; three trials showed that compared to chemotherapy alone, herbal formulas plus chemotherapy may increase the KPS score (Yang et al., 2008, Jia and Dong, 2019, Xue et al., 2021); another trial reported no significant difference in KPS score between two groups (Liu, 2016). The meta-analysis for these two outcomes was not conducted owing to the existence of considerable heterogeneity. One trial showed that herbal formulas plus FOLFOX chemotherapy may increase the QLQ-C30 score by 6.93 points as compared to FOLFOX chemotherapy alone (Jia and Dong, 2019). The meta-analysis of three trials showed that herbal formulas plus chemotherapy may help to increase the KPS improvement rate by 62% [RR = 1.62, 95% CI (1.13, 2.32)] (Nan and Li, 2016, Zhang et al., 2018; Wang and Zhang, 2018).

##### 3.3.3.4 Safety outcomes for advanced colorectal cancer

**Figure 11:**
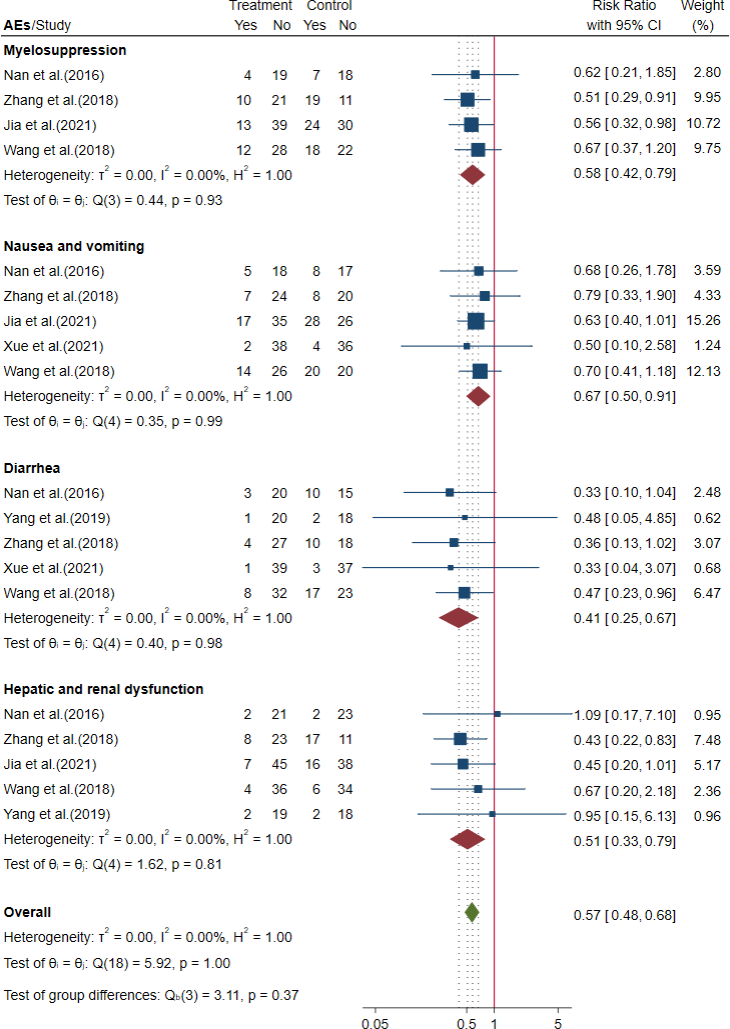
Forest plot of incidence of adverse events (AEs) in advanced colorectal cancer (CRC).

Six trials reported the incidence of AEs in advanced CRC (Wang and Zhang, 2018; Yang et al., 2019, Nan and Li, 2016, Zhang et al., 2018, Jia and Dong, 2019, Xue et al., 2021). The meta-analysis showed that compared to chemotherapy alone, herbal formulas plus chemotherapy could decrease the incidence of myelosuppression by 42% [RR = 0.58, 95% CI (0.42, 0.79)], nausea and vomiting by 33% [RR = 0.67, 95% CI (0.50, 0.91)], diarrhea by 59% [RR = 0.41, 95% CI (0.25, 0.67)], and hepatic and renal dysfunction by 49% [RR = 0.51, 95% CI (0.33, 0.79)]. The results are shown in **Figure 11** and **Table 3**.

### 3.4 Publication bias

Analysis of publication bias was not implemented since the studies included in separate subgroups were less than 10.

### 3.5 Quality of evidence assessment

We assessed 21 synthesized pieces of evidence with GRADE. A total of 14 of these outcomes were assessed as low certainty, and seven were very low certainty. The main reasons to downgrade the quality of evidence are the unsatisfactory risk of bias and the limited sample size of included trials. The summary of findings is shown in **Table 4**.

**Table 4:** Summary of findings.

Outcome No. of participants(studies)	Relative effect(95% CI)	Anticipated absolute effects (95% CI)			Certainty
		Without herbal medicine	With herbal medicine	Difference	
Perioperative gastric cancer					
Time to bowel sound recovery (herbal medicine + ERAS *vs*. ERAS) No. of participants: 120 (2 RCTs)	–	**Mean: 20.03** h	-	MD **1.63 h shorter** (2.62 fewer to 0.65 fewer)	□□○○ Low^a,b^
Time to first flatus (herbal medicine + EN *vs*. EN) No. of participants: 200 (2 RCTs)	–	**Mean: 31.20** h	-	MD **9.69** h **shorter** (10.89 fewer to 8.48 fewer)	□□○○ Low^a,b^
Time to first defecation (herbal medicine + EN *vs*. EN) No. of participants: 200 (2 RCTs)	–	**Mean: 30.15** h	-	MD **0.72** h **shorter** (1.68 fewer to 0.25 more)	□□○○ Low^a,b^
Time to first oral feeding (herbal medicine + EN *vs*. EN) No. of participants: 200 (2 RCTs)	–	**Mean: 40.63** h	-	MD **2.74** h **shorter** (3.94 fewer to 1.54 fewer)	□□○○ Low^a,b^
Time to out-of-bed activity (herbal medicine + EN *vs*. EN) No. of participants: 120 (2 RCTs)	–	**Mean: 12.84** h	-	MD **2.11** h **shorter** (3.04 fewer to 1.19 fewer)	□□○○ Low^a,b^
Duration of hospitalization (herbal medicine + ERAS *vs*. ERAS) No. of participants: 204 (2 RCTs)	–	**Mean: 13.85** days	-	MD **2.09 days fewer** (2.75 fewer to 1.43 fewer)	□□○○ Low^a,b^
Duration of hospitalization (herbal medicine + EN *vs*. EN) No. of participants: 200 (2 RCTs)	–	**Mean: 16.52** days	-	MD **4 days fewer** (5.16 fewer to 2.84 fewer)	□□○○ Low^a,b^
Advanced gastric cancer					
ORR (herbal medicine + FOLFOX *vs*. FOLFOX) No. of participants: 172 (2 RCTs)	**RR 1.67** (1.21 to 2.30)	38.4%	**64.1%** (46.4 to 88.3)	**25.7% more** (8.1 more to 49.9 more)	□□○○ Low^a,b^
ORR (herbal medicine + 5-FU+CF+paclitaxel) No. of participants: 151 (2 RCTs)	**RR 1.03** (0.79 to 1.33)	59.5%	**61.2%** (47 to 79.1)	**1.8% more** (12.5 fewer to 19.6 more)	□○○○ Very low^a,b,c^
ORR (herbal medicine + other chemotherapy *vs*. other chemotherapy) No. of participants: 330 (3 RCTs)	**RR 1.40** (1.18 to 1.66)	47.9%	**67.0%** (56.5 to 79.5)	**19.2% more** (8.6 more to 31.6 more)	□○○○ Very low^a,b,d^
DCR (herbal medicine + FOLFOX *vs*. FOLFOX) No. of participants: 172 (2 RCTs)	**RR 1.32** (1.11 to 1.58)	65.1%	**86.0%** (72.3 to 100)	**20.8% more** (7.2 more to 37.8 more)	□□○○ Low^a,b^
DCR (herbal medicine + 5-FU+CF+paclitaxel) No. of participants: 151 (2 RCTs)	**RR 0.96** (0.82 to 1.13)	81.1%	**77.8%** (66.5 to 91.6)	**3.2% fewer** (14.6 fewer to 10.5 more)	□□○○ Low^a,b^
ORR (herbal medicine + other chemotherapy *vs*. other chemotherapy) No. of participants: 330 (3 RCTs)	**RR 1.10** (1.02 to 1.18)	78.8%	**86.7%** (80.4 to 93)	**7.9% more** (1.6 more to 14.2 more)	□○○○ Very low^a,b,d^
KPS improvement rate (herbal medicine + 5-FU+CF+paclitaxel *vs*. 5-FU+CF+paclitaxel) No. of participants: 129 (2 RCTs)	**RR 1.56** (1.17 to 2.09)	45.3%	**70.7%** (53 to 94.7)	**25.4% more** (7.7 more to 49.4 more)	□□○○ Low^a,b^
Perioperative colorectal cancer					
Time to first flatus (herbal medicine + SC *vs*. SC) No. of participants: 153 (3 RCTs)	–	**Mean: 56.94** h	-	MD **3.26** h **shorter** (13.75 fewer to 7.23 more)	□□○○ Low^b,e^
Postoperative colorectal cancer					
Mean PFS (Quxie Capsule *vs*. control) No. of participants: 140 (2 RCTs)	–	**Mean: 17.28** months	-	MD **8.7 months more** (3.27 more to 14.13 more)	□□○○ Low^b,e^
KPS improvement rate (herbal medicine + chemotherapy *vs*. chemotherapy) No. of participants: 416 (4 RCTs)	**RR 1.96** (1.38 to 2.79)	16.9%	**33.1%** (23.3 to 47.2)	**16.2% more** (6.4 more to 30.3 more)	□□○○ Low^a,d^
Advanced colorectal cancer					
ORR (herbal medicine + FOLFOX *vs*. FOLFOX) No. of participants: 306 (3 RCTs)	**RR 1.44** (0.92 to 2.25)	35.1%	**50.5%** (32.3 to 78.9)	**15.4% more** (2.8 fewer to 43.8 more)	□○○○ Very low^a,b,c,e^
ORR (herbal medicine + RTX-based chemotherapy *vs*. RTX-based chemotherapy) No. of participants: 105 (2 RCTs)	**RR 1.02** (0.57 to 1.81)	29.4%	**30.0%** (16.8 to 53.2)	**0.6% more** (12.6 fewer to 23.8 more)	□○○○ Very low^a,b,c^
DCR (herbal medicine + RTX-based chemotherapy *vs*. RTX-based chemotherapy) No. of participants: 105 (2 RCTs)	**RR 1.09** (0.89 to 1.34)	74.5%	**81.2%** (66.3 to 99.8)	**6.7% more** (8.2 fewer to 25.3 more)	□○○○ Very low^a,b,c^
KPS improvement rate (herbal medicine + chemotherapy *vs*. chemotherapy) No. of participants: 187 (3 RCTs)	**RR 1.62** (1.13 to 2.32)	29.0%	**47.0%** (32.8 to 67.4)	**18.0% more** (3.8 more to 38.3 more)	□○○○ Very low^a,b,c^

CI, confidence interval; MD, mean difference; ERAS, enhanced recovery after surgery; RCTs, randomized controlled trials; EN, enteral nutrition; ORR, objective response rate; DCR, disease control rate; KPS, Karnofsky performance status; PFS, progression-free survival.

a All studies were assessed as having ‘Some concerns’ risk of bias.

b Small study sample size.

c 95% CI overlaps no effect (RR of 1.0).

d Clinical heterogeneity exists.

e Statistical heterogeneity exists, I^2^ > 50%.

The bold was generated in the original form of the SoF table in the GRADE system.

## 4 Discussion

In this study, after pre-searching classical Chinese herbal prescriptions and Chinese patent medicines that regulate gut microbiota, we assessed the efficacy and safety of HFGMR in GC and CRC. 1) In perioperative stages, HFGMR could promote the recovery of gastrointestinal function, shorten the time of hospital stays, improve the QoL in the perioperative period of GC and CRC, and not increase the extra incidence of AEs. 2) In the postoperative stage with adjuvant chemotherapy, HFGMR can improve the QoL of patients and reduce the incidence of nausea and vomiting in both GC and CRC. Furthermore, HFGMR could significantly prolong DFS in CRC and reduce the incidence of anorexia, diarrhea, and peripheral neurotoxicity in GC and leucopenia in CRC. However, a significant difference in long-term survival efficacy for GC was not observed. 3) In the advanced stage, because there is a large heterogeneity of the reported studies in long-term survival, we mainly focus on the TRR. The combination of chemotherapy (except 5-FU+CF+paclitaxel regimen) and HFGMR could significantly improve ORR and DCR in GC. However, it is a pity that there was no significant difference between HFGMR plus chemotherapy and chemotherapy in ORR and DCR in CRC, which might be related to the small number of included studies and sample size. HFGMR plus chemotherapy can improve the QoL and reduce the occurrence of gastrointestinal reactions and myelosuppression. It is worth proposing that HFGMR can improve fatigue in GC and liver and kidney function in CRC.

Several herbal formulas showed their function to regulate the gut microbiota (Shao et al., 2021), but the relationship between the function of regulating gut microbiota and anti-tumor efficacy remains unclear. Danggui Buxue decoction and Liujunzi decoction were reported to downregulate *Lactobacillus*, which was considered a probiotic in the past, may suppress inflammatory T-cell infiltration and promote tumor growth in pancreatic cancer (Shi et al., 2021, Cheng et al., 2021a; Hezaveh et al., 2022). Herbal formulas exerted anti-cancer efficacy through multiple mechanisms and pathways, and regulation of gut microbiota requires more attention. Previous studies showed that herbal formulas can improve the TRR, QoL, peripheral blood immune cell function, and fatigue status (Huang et al., 2020); reduce the incidence of AEs (Chen et al., 2018; Lu et al., 2021); and improve 1- and 2-year survival rates and the occurrence of liver dysfunction, renal dysfunction, neurotoxicity, and alopecia in gastrointestinal cancer patients, which is consistent with our study (Cheng et al., 2021b). Interestingly, a previous study evaluating the efficacy of herbal formulas combined with paclitaxel-based chemotherapy in GC found that the combination therapy could significantly improve the TRR [ORR: 1.39; 95% CI (1.24, 1.57), I^2^ = 12%], and the small sample size was the disadvantage (Li et al., 2020c). Inconsistent with this study, it was reported that herbal formulas combined with chemotherapy can improve the TRR in CRC patients but have no improvement effect on liver and kidney dysfunction (Lin et al., 2019). The reason for this discrepancy between the two studies may be that the trials in our study have a smaller sample size and selection bias. It was found in another study that 5-fluorouracil-based chemotherapy combined with herbal formulas has more effect in improving the TRR in patients with CRC (Chen et al., 2019). As the only Chinese patent medicine included in this meta-analysis, Quxie Capsule was reported in a study to have a good effect on reducing the 1- and 2-year recurrence and metastasis rate and relieving symptoms in CRC, which provide us more information on the long-term effect of HFGMR (Zhang et al., 2021b). However, their quality is low and needs to be confirmed by more high-quality clinical studies.

Although we strictly conducted this meta-analysis according to the review procedure released by the Cochrane Collaboration, this study has several limitations. First, in spite of the definite effect of HFGMR according to the previous studies, only one study reported the results of gut microbiota after medication intervention among the included clinical studies in the meta-analysis. Furthermore, OS and PFS, the main indicators to evaluate the long-term efficacy of anti-tumor treatment, have not been monitored, which makes the long-term effects of HFGMR in GC and CRC remain unknown. Moreover, high-quality original studies were scarce in this study. The problems in most RCTs included a low utilization rate of blinding and unreported lost follow-up cases. Finally, age, gender, race, culture, and diet, as well as geographical location, are the main factors to influence gut microbiota, which have not been reported and considered in this study.

Due to the limitations associated with the poor quality of pooled studies, it is difficult to draw a definitive conclusion. Nevertheless, our study suggests the positive effect of HFGMR in facilitating the management of duration of hospitalization, ORR, DCR and KPS, and AEs in perioperative, postoperative, and advanced GC patients. HFGMR can also improve the PS in postoperative patients with CRC, which might be a positive strategy against GC and CRC, and provides a new therapeutic option in clinical management. In future clinical trials (randomized, double-blind, and placebo-controlled design), factors impacting gut microbiota should be fully considered in the design and implementation process.

## 5 Conclusion

This study indicates that herbal formulas, which could regulate the composition and proportion of gut microbiota, have a positive effect in three stages (perioperative, postoperative, and advanced) of GC and CRC. They could promote the recovery of postoperative gastrointestinal function, increase TRR, improve KPS, and reduce the incidence of AEs. Herbal formulas exert anti-cancer efficacy through multiple mechanisms and pathways; among them, the regulation of gut microbiota has not been paid enough attention. To further support the conclusion and better understand the role of gut microbiota in the treatment of GC and CRC, more rigorously designed, large-scale, and multicenter RCTs that focus on herbal formulas and gut microbiota are needed in the future.

## Author contributions

HW and XW conceived this study. HW and LC registered the protocol. BX and HW performed the search, screen, inclusion, and quality assessment of the included trials. BX and HW conducted the meta-analysis. BX, HW, XW, and YG drafted the first version of this manuscript. BY and RG provided critical revisions and edited the manuscript. JL revised the manuscript. All authors read and approved the final manuscript for submission.

## Funding

This study was supported by the China Academy of Chinese Medical Sciences (CACMS) Innovation Fund (Grant no. CI2021A01802).

## Conflict of interest

The authors declare that the research was conducted in the absence of any commercial or financial relationships that could be construed as a potential conflict of interest.

## Publisher’s note

All claims expressed in this article are solely those of the authors and do not necessarily represent those of their affiliated organizations, or those of the publisher, the editors and the reviewers. Any product that may be evaluated in this article, or claim that may be made by its manufacturer, is not guaranteed or endorsed by the publisher.
