# Supplementary material for: Efficacy and safety of herbal formulas with the function of gut microbiota regulation for gastric and colorectal cancer: A systematic review and meta-analysis
Source: Front Cell Infect Microbiol. 2022 Aug 4;12:875225. doi: 10.3389/fcimb.2022.875225 (PMC9386000; doi:10.3389/fcimb.2022.875225)
Supplement: Supplementary file 1 [file DataSheet_1.docx]

**Search strategy used in Pubmed**

#1 (("Stomach Neoplasms"[Mesh]) OR (((((((((((Neoplasm, Stomach[Title/Abstract]) OR (Stomach Neoplasm[Title/Abstract])) OR (Neoplasms, Stomach[Title/Abstract])) OR (Gastric Neoplasms[Title/Abstract])) OR (Gastric Neoplasm[Title/Abstract])) OR (Neoplasm, Gastric[Title/Abstract])) OR (Neoplasms, Gastric[Title/Abstract])) OR (Cancer of Stomach[Title/Abstract])) OR (Stomach Cancers[Title/Abstract])) OR (Gastric Cancer[Title/Abstract])) OR (Cancer, Gastric[Title/Abstract])) OR (Cancers, Gastric[Title/Abstract])) OR (Gastric Cancers[Title/Abstract]) OR (Stomach Cancer[Title/Abstract]) OR (stomach tumor[Title/Abstract]) OR (Cancer, Stomach[Title/Abstract]) OR (Cancers, Stomach[Title/Abstract]) OR (Cancer of the Stomach[Title/Abstract]) OR (Gastric Cancer, Familial Diffuse[Title/Abstract])

#2 (("Colorectal Neoplasms"[Mesh]) OR (((((((((((Colorectal Neoplasm[Title/Abstract]) OR (Neoplasm, Colorectal[Title/Abstract])) OR (Neoplasms, Colorectal[Title/Abstract])) OR (Colorectal Tumors[Title/Abstract])) OR (Colorectal Tumor[Title/Abstract])) OR (Tumor, Colorectal[Title/Abstract])) OR (Tumors, Colorectal[Title/Abstract])) OR (Colorectal Cancer[Title/Abstract])) OR (Cancer, Colorectal[Title/Abstract])) OR (Cancers, Colorectal[Title/Abstract])) OR (Colorectal Cancers[Title/Abstract])) OR (Colorectal Carcinoma[Title/Abstract])) OR (Carcinoma, Colorectal[Title/Abstract]) OR (Carcinomas, Colorectal[Title/Abstract]) OR (Colorectal Carcinomas[Title/Abstract])

#3 (("Colonic Neoplasms"[Mesh]) OR (((((((((((Colonic Neoplasm[Title/Abstract]) OR (Neoplasm, Colonic[Title/Abstract])) OR (Neoplasms, Colonic[Title/Abstract])) OR (Colon Neoplasms[Title/Abstract])) OR (Colon Neoplasm[Title/Abstract])) OR (Neoplasm, Colon[Title/Abstract])) OR (Neoplasms, Colon[Title/Abstract])) OR (Cancer of Colon[Title/Abstract])) OR (Colon Cancers[Title/Abstract])) OR (Colon Cancer[Title/Abstract])) OR (Colon tumor[Title/Abstract])) OR (Cancer, Colon[Title/Abstract])) OR (Cancers, Colon[Title/Abstract]) OR (Cancer of the Colon[Title/Abstract]) OR (Colonic Cancer[Title/Abstract]) OR (Cancer, Colonic[Title/Abstract]) OR (Cancers, Colonic[Title/Abstract]) OR (Colonic Cancers[Title/Abstract]) OR (Colon Adenocarcinoma[Title/Abstract]) OR (Adenocarcinoma, Colon[Title/Abstract]) OR Adenocarcinomas, Colon[Title/Abstract] OR Colon Adenocarcinomas[Title/Abstract]

#4 (("Rectal Neoplasms"[Mesh]) OR (((((((((((Neoplasm, Rectal[Title/Abstract]) OR (Rectal Neoplasm[Title/Abstract])) OR (Rectum Neoplasms[Title/Abstract])) OR (Neoplasm, Rectum[Title/Abstract])) OR (Rectum Neoplasm[Title/Abstract])) OR (Rectal Tumors[Title/Abstract])) OR (Rectal Tumor[Title/Abstract])) OR (Tumor, Rectal[Title/Abstract])) OR (Neoplasms, Rectal[Title/Abstract])) OR (Cancer of Rectum[Title/Abstract])) OR (Rectum Cancers[Title/Abstract])) OR (Rectal Cancer[Title/Abstract])) OR (Cancer, Rectal[Title/Abstract]) OR (Rectal Cancers[Title/Abstract]) OR (Rectum Cancer[Title/Abstract]) OR (Cancer, Rectum [Title/Abstract]) OR (Cancer of the Rectum[Title/Abstract])

#5 #1 OR #2 OR #3 OR #4

#6 ((Sijunzi decoction[Supplementary Concept]) OR (si-jun-zi-tang[Supplementary Concept]) OR (SJZD[Title/Abstract])) OR (Sijunzi[Title/Abstract]) OR (Sijunzi formula[Title/Abstract])

#7 (((gegenqinlian[Supplementary Concept]) OR (gegen qinlian[Title/Abstract])) OR (gegenqinlian decoction[Title/Abstract])) OR (gegenqinlian formula[Title/Abstract])

#8 ((liujunzi[Supplementary Concept]) OR (liu-jun-zi-tang[Supplementary Concept]) OR (liujunzi decoction[Title/Abstract])) OR (rikkunshi-to[Supplementary Concept]) OR (rikkunshito[Title/Abstract]) OR (TJ-43[Supplementary Concept]) OR (liujunzi formula[Title/Abstract])

#9 (((((((((((xiaochaihu[Supplementary Concept]) OR (xiaochaihu decoction[Title/Abstract])) OR (xiaochaihu formula[Title/Abstract])) OR (shosaiko-to [Supplementary Concept])) OR (shosaiko-toh[Title/Abstract])) OR (sho-saiko-to[Title/Abstract])) OR (xiaochaihutang[Title/Abstract])) OR (XCHT herbal formula[Title/Abstract])) OR (xiao-chai-hu-tang[Title/Abstract])) OR (xiaochaihu-tang[Title/Abstract])) OR (TJ-9[Title/Abstract])) OR (TJ9[Title/Abstract])

#10 ((((danggui buxue decoction [Supplementary Concept]) OR (dangguibuxue decoction[Title/Abstract])) OR (dangguibuxue formula[Title/Abstract])) OR (danggui buxue[Title/Abstract])) OR (Dang-Gui-Bu-Xue decoction[Title/Abstract]) OR (Danggui Buxue Tang[Title/Abstract])

#11 ((shenling baizhu san [Supplementary Concept]) OR (shenling baizhu decoction[Title/Abstract])) OR (shenling baizhu formula[Title/Abstract])

#12 ((((dai-kenchu-to [Supplementary Concept]) OR (dai-kenchu-to[Title/Abstract])) OR (daikenchuto[Title/Abstract])) OR (da jian zhong tang[Title/Abstract])) OR (dajianzhong[Title/Abstract]) OR (TU-100 extract[Title/Abstract])

#13 (Jishengwumeipian[Title/Abstract]) OR (quxie capsule[Title/Abstract])

#14 #6 OR #7 OR #8 OR #9 OR #10 OR #11 OR #12 OR #13

#15 #5 AND #14

#16 ((randomized controlled trial[pt] OR controlled clinical trial[pt] OR randomized[tiab] OR placebo[tiab] OR clinical trials as topic[mesh:noexp] OR randomly[tiab] OR trial[ti] NOT (animals[mh] NOT humans [mh])))

#17 #5 AND #14 AND #16
